# MZF1 Inhibits TGFβ‐1/SMAD Signaling by Transcriptionally Activating SnoN to Augment Tumorigenic Potential in Colon Cancer

**DOI:** 10.1002/advs.77277

**Published:** 2026-10-20

**Authors:** Siqi Sheng, Mengxi Huang, Yiting Wang, Hongyu Wang, Haimei Feng, Xiaoming Bai, Jie Chen, Ke Xu, Xiaoyuan Chu, Zengjie Lei

**Affiliations:** ^1^ Department of Medical Oncology Medical School Affiliated Jinling Hospital Nanjing University Nanjing Jiangsu Province China; ^2^ Department of Medical Oncology Jinling Clinical Medical College Nanjing University of Chinese Medicine Nanjing Jiangsu Province China; ^3^ Department of Medical Oncology Jinling Hospital Nanjing Medical University Nanjing Jiangsu Province China; ^4^ Department of Medical Oncology The First School of Clinical Medicine Jinling Hospital Southern Medical University Nanjing Jiangsu Province China; ^5^ Department of Oncology School of Medicine Affiliated Jinling Hospital Southeast University Nanjing China

**Keywords:** colon cancer, epigenetic regulation, MZF1, proliferation, SnoN, transcriptional activation

## Abstract

Colon cancer progression is associated with transcriptional and signaling abnormalities. MZF1, linked to leukemogenesis, has an unclear role in colon cancer. This study examined its function in colon tumorigenesis and signaling‐targeted therapies. Multi‐omics (RNA‐seq, CUT&Tag) identified MZF1 targets, validated in vitro/in vivo using xenografts and epithelial‐specific MZF1 knockout mice. Virtual screening found MZF1 inhibitors. MZF1 activated SnoN transcription, suppressing TGF‐β/SMAD signaling. Overexpression enhanced proliferation and colony formation; knockdown reversed these. In vivo, MZF1‐overexpressing xenografts grew faster with more Ki67+ cells, while MZF1^−/−^ mice had reduced tumor burden. Organoids from MZF1^−/−^ mice grew poorly. 10Panx Acetate, an MZF1 inhibitor, disrupted MZF1‐SnoN interaction, inhibiting cell and xenograft growth. Our results indicate MZF1 as a key colon cancer regulator via SnoN‐mediated TGF‐β pathway suppression and suggest 10Panx Acetate for precision therapy. MZF1/SnoN co‐expression may predict aggressive disease.

## Graphical abstract

This study identifies MZF1 as a core regulator of colon cancer via SnoN‐mediated TGF‐β suppression, proposes 10Panx Acetate as a targeted inhibitor, and validates MZF1/SnoN as prognostic markers for aggressive disease.

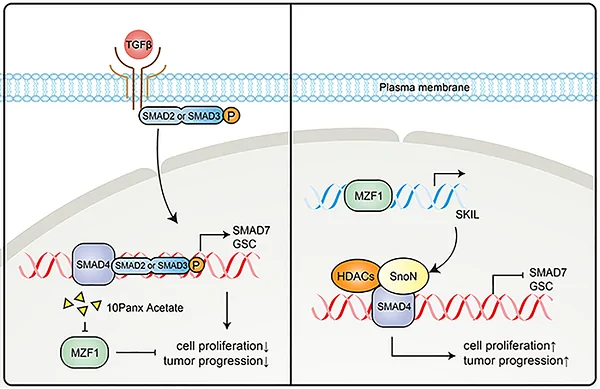

## 1 Introduction

Colorectal cancer (CRC) remains a leading cause of cancer‐related mortality worldwide, with over 1.9 million new cases and 904 000 deaths in 2022 [1]. Despite recent advances in the diagnosis and treatment of CRC, such as improvements in surgical techniques, the development of chemotherapeutic drugs, and the application of targeted therapies, the overall prognosis for patients remains poor, especially for those with advanced CRC who are prone to recurrence and metastasis and have low survival rates [2, 3, 4]. Therefore, in‐depth exploration of the molecular mechanisms underlying the initiation and progression of CRC, and the identification of new therapeutic targets, are necessary for improving the treatment outcomes of CRC patients. Accumulating evidence highlights the critical role of transcriptional regulation in the pathogenesis of CRC [5, 6, 7]. However, the molecular mechanisms behind these processes remain poorly understood.

Transcription factors (TFs) are a class of proteins that can bind to specific DNA sequences to regulate gene expression. They play crucial roles in various biological processes, such as cell growth, differentiation, and apoptosis. Abnormal expression or activity changes of TFs are often closely associated with the occurrence and development of tumors [2, 3, 4]. TFs can influence malignant biological behaviors such as cell proliferation, migration, and invasion by activating or inhibiting the expression of downstream target genes, thereby participating in tumor formation and progression. Therefore, transcription factors are promising therapeutic targets for cancer therapy [8].

Myeloid Zinc Finger 1 (MZF1) belongs to the zinc finger transcription factor subfamily. Previous studies have shown that MZF1 is abnormally expressed in various tumors, and its gene copy number is significantly altered in breast cancer, lung cancer, bladder cancer, glioma, and colon cancer [9, 10]. Recent studies indicate that MZF1 participates in carcinogenesis through diverse mechanisms. In liver cancer, MZF1 accelerates PD‐L1 ubiquitination by binding to the CDK4 activation site, promoting immune escape [11]. In gastric cancer, MZF1 acts as an upstream transcription factor of miR‐328‐3p and inhibits tumor progression by activating the miR‐328‐3p/CD44 axis [12]. In prostate cancer, MZF1 interacts with the SCAND1 protein and inhibits the epithelial‐mesenchymal transition (EMT) and tumor metastasis through chromatin remodeling [13]. However, the specific mechanism of MZF1 in CRC has not been fully elucidated, and its regulatory network during the initiation and progression of CRC as well as its interactions with other signaling pathways, remain to be thoroughly investigated.

Therefore, we set out to determine the role of MZF1 in CRC, aiming to reveal the molecular mechanisms by which it regulates CRC initiation and progression through transcriptional activation of downstream target genes, providing a theoretical basis and potential targets for CRC treatment. Here, we report a previously unrecognized mechanism by which MZF1 drives CRC progression through direct transcriptional activation of SnoN, leading to inhibition of the TGF‐β/SMAD signaling pathway. Using integrated multi‐omics approaches, including RNA sequencing, CUT&Tag, and ChIP‐qPCR, we demonstrate that MZF1 binds to the SnoN promoter to enhance its expression, thereby inhibiting the phosphorylation of SMAD2/3 and abrogating TGF‐β‐induced growth arrest. Furthermore, we validate the therapeutic potential of targeting the MZF1/SnoN axis using a small‐molecule inhibitor, 10Panx Acetate, which promotes the degradation of MZF1, thereby downregulating SnoN expression and suppressing tumor growth in preclinical models. Therefore, MZF1‐SnoN‐TGF‐β is a novel signaling axis in CRC and targeting this pathway represents a promising therapeutic target for precision medicine‐based CRC treatment.

## 2 Results

### 2.1 MZF1 is a Critical Transcription Factor Controlling CRC Malignant Progression

To identify epigenetic regulatory factors involved in CRC development, we first performed bioinformatics analysis on GEO mRNA expression profiling datasets. A Venn diagram revealed 10,135 genes commonly present across three platforms (GPL570, GPL5175, GPL96) in 1215 CRC and adjacent normal tissues (Figure S1A and Table S4). The volcano plot identified 514 genes with significantly higher expression (*p* < 0.05) and 545 genes with significantly lower expression (*p* < 0.05) in tumor tissues compared to normal tissues (Figure 1B). We selected the top 100 upregulated and downregulated differentially expressed genes (DEGs) for heat map visualization (Figure 1A and Table S5). Considering the complexity of tumorigenesis through transcriptional co‐regulation networks, we performed transcriptional co‐regulatory analysis on these 200 DEGs and identified four top transcriptional factors: SPI1, MZF1, SOX10, and NFIC (Figure 1C and Table S6). These findings suggest that these transcriptional factors may play important roles in CRC development and progression.

**FIGURE 1:**
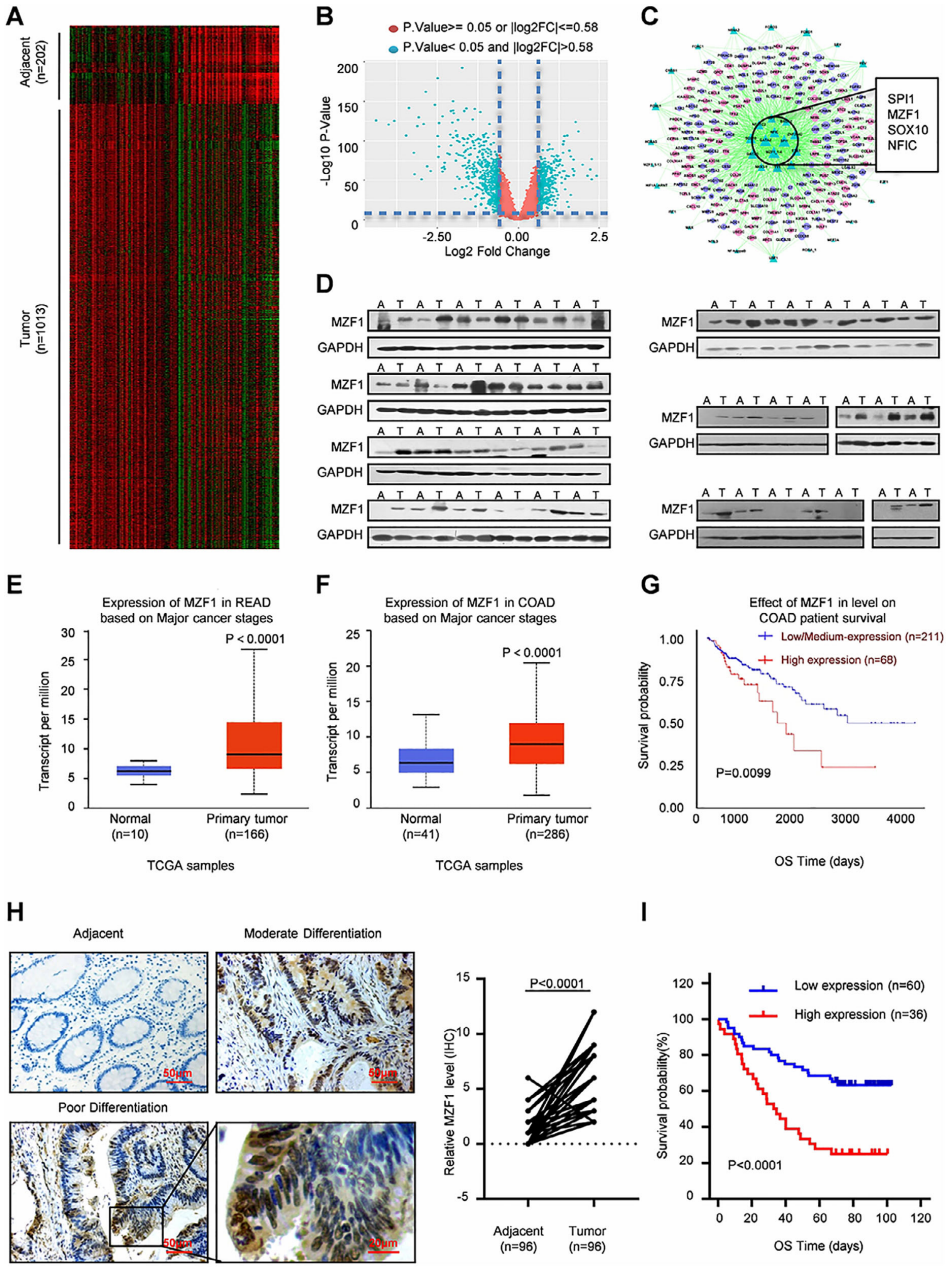
MZF1 as a critical transcription factor involved in the malignant progression of colorectal cancer. (A) Heatmap of the top 100 upregulated and downregulated differentially expressed genes between tumor tissue and normal tissue in colorectal cancer mRNA expression profile chip. The horizontal axis represents 200 differentially expressed genes, and the vertical axis represents each tissue sample. The green to black to red color in the figure represents the gradual increase of gene expression levels (standardized values) from −1 to 0 to 1. (B) Volcano plot of mRNA expression profiles from 1013 colorectal cancer tumor tissues and 202 adjacent tissues in GEO database. Green: Differential genes with FC>1.5 and *p* < 0.05 between tumor tissue and adjacent tissue; Red: Non‐differential genes with FC≤1.5 or ≥0.6 or *p* ≥ 0.05 between tumor tissue and adjacent tissue. FC, fold change. (C) Transcriptome analysis network diagram of differentially expressed genes. The top four transcription factors with the highest scores are listed in the box (SPI1, MZF1, SOX10, and NFIC). (D) Immunoblotting of MZF1 in tumor tissue (T) and normal adjacent tissue (A) of clinical specimens. GAPDH is used as an internal reference. (E) mRNA expression of MZF1 in COAD from TCGA datasets. (F) mRNA expression of MZF1 in READ from TCGA datasets. (G) Analysis of MZF1 expression levels and patient prognosis in TCGA database. Blue, MZF1 low expression group (*n* = 68); red, MZF1 high expression group (*n* = 211). (H) Immunohistochemical (IHC) analysis of MZF1 expression levels in tumor tissue and normal tissue, with brown staining indicating positive staining. Statistical analysis of MZF1 expression levels in tumor tissue and corresponding normal tissue (*n* = 96). (I) Kaplan–Meier curve of MZF1 expression level in tumor tissue and patient OS prognosis. (high: *n* = 36, low: *n* = 60).

We verified protein expression levels of these five factors using immunoblotting in 11 clinical tissue pairs. MZF1 exhibited significant overexpression in tumor tissues, while SOX10, NFIC, and SPI1 showed no marked differences, or where inconsistent, between tumor and normal tissues (Figure 1D and Figure S1D). Subsequent analysis of 30 additional clinical samples confirmed that MZF1 protein levels were significantly elevated in CRC tissues compared to matched normal tissues (Figure 1D).

Multi‐platform analysis of TCGA datasets further demonstrated that MZF1 mRNA expression was significantly upregulated in both rectal (Figure 1E) and colon (Figure 1F) cancer tissues compared to normal counterparts (*p* < 0.001). Furthermore, MZF1 expression correlated with poor prognosis in CRC patients (Figure 1G). Immunohistochemical analysis of 96 clinical specimens confirmed increased MZF1 expression in CRC tumor tissues compared to adjacent tissues with a predominantly nuclear localization (Figure 1H). Segregation of tumor tissues based on differentiation grade suggested a trend toward higher MZF1 expression in lower‐grade tumors; however, this difference did not reach statistical significance (*p* = 0.056; Table S6). Kaplan–Meier survival analysis of patients dichotomized by the median MZF1 immunohistochemical score (MZF1‐high vs. MZF1‐low) revealed significantly shorter overall survival (OS), disease‐free survival (DFS), and metastasis‐free Survival (MFS) in patients with high MZF1 expression (*p* < 0.05; Figure 1I and Figure S1B,C). Multivariate Cox regression analysis identified high MZF1 expression as an independent risk factor for overall survival (HR = 2.083, 95% CI: 1.142–3.802; *p* = 0.017) (Tables S7 and S8). Collectively, these data establish MZF1 as a critical transcriptional regulator promoting malignant progression in CRC, where its overexpression associates with adverse clinical outcomes.

### 2.2 MZF1 Promotes Colon Cancer Cell Proliferation In Vitro

To investigate the biological function of MZF1 in colon cancer, we first analyzed baseline MZF1 expression levels across multiple colon cancer cell lines. Compared with normal colon epithelial cells, MZF1 was significantly upregulated in colon cancer cells (Figure S2A). Notably, SW480 and HCT8 exhibited relatively low MZF1 expression, whereas CACO2 and HCT15 showed high MZF1 expression. Based on this, we constructed stable MZF1‐overexpressing cell lines (SW480, HCT8) and verified overexpression efficiency via qPCR and immunoblotting (Figure 2A,B and Figure S2B,C). Conversely, we generated MZF1‐depleted cell lines (CACO2, HCT15) and confirmed knockdown efficiency as above (Figure 2C,D and Figure S2D,E). Using these engineered cell lines, we systematically investigated the functional role of MZF1 in colon cancer. Using the CCK8 assay, we found that MZF1 overexpression enhanced colon cancer cell proliferation in both lines (Figure 2E,F). This enhanced proliferation was further confirmed using colony formation and soft agar assays (Figure 2G–L and Figure S2F,G). However, transwell migration assays revealed no significant impact of MZF1 overexpression on the migratory ability of HCT8 and SW480 cells (Figure S2H,I). Depleting MZF1 in CACO2 and HCT15 colon cancer cells resulted in reduced proliferation in CCK8, colony formation, and soft agar assays (Figure 2M–R and Figure S2J,K). Collectively, these in vitro experiments establish that MZF1 overexpression enhances, while MZF1 deficiency suppresses, colon cancer cell proliferation.

**FIGURE 2:**
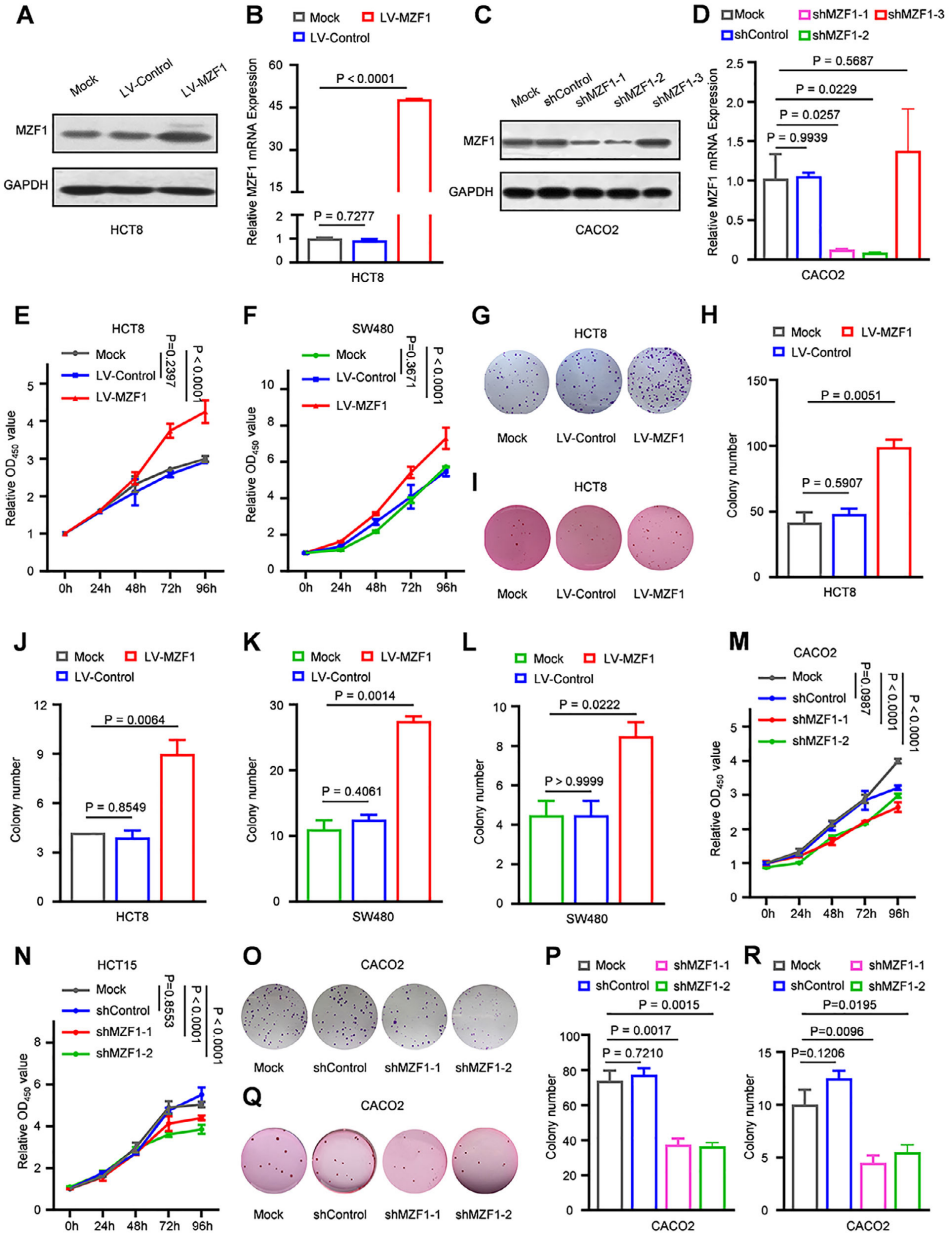
MZF1 promotes colon cancer cell proliferation in vitro. (A,B) Protein (A) and mRNA (B) levels of MZF1 in HCT8 cell lines infected with control or MZF1 overexpression lentivirus and mock‐infected cells using immunoblotting (A) and RT‐qPCR (B). (C,D) Protein (C) and mRNA (D) levels of MZF1 in CACO2 cells infected with control or MZF1 shRNA lentivirus and mock cells using immunoblotting (C) and RT‐qPCR (D). (E,F) CCK8 of HCT116 (E) and SW480 (F) cells infected with control or MZF1 overexpression lentivirus and mock cells. (G,H) Representative images (G) and quantification (H) of colony formation assays of HCT8 cells infected with control or MZF1 overexpression lentivirus and mock‐infected cells. (I,J) Representative images (I) and quantification (J) of soft agar assays of HCT8 cells infected with control or MZF1 overexpression lentivirus and mock‐infected cells. (K) Quantification of colony formation assays of SW480 cells infected with control or MZF1 overexpression lentivirus and mock‐infected cells. (L) Quantification of soft agar assay of SW480 cells infected with control or MZF1 overexpression lentivirus and mock‐infected cells. (M,N) CCK8 of CACO2 (M) and HCT15 (N) cells infected with shControl or shMZF1 expressing lentivirus and mock infected cells. (O,P) Representative images (O) and quantification (P) of colony formation assays in CACO2 cells infected with shControl or shMZF1‐expressing lentivirus and mock‐infected cells. (Q,R) Representative images (Q) and quantification (R) of soft agar assays in CACO2 cells infected with shControl or shMZF1‐expressing lentivirus and mock‐infected cells. All assays were performed in triplicate and *p* values were calculated using two‐tailed, unpaired Student's *t*‐test.

### 2.3 MZF1 Plays an Important Role in Promoting the Progression of Colon Cancer In Vivo

To further investigate the pro‐tumorigenic function of MZF1 in colon cancer, we conducted in vivo xenograft studies using stably luciferase‐expressing MZF1‐overexpressing cells (Fluc‐HCT8‐LvMZF1) and control cells (Fluc‐HCT8‐Lvcontrol). Each of these lines were subcutaneously engrafted into Nude mice and live imaging was performed weekly to monitor tumor volume and fluorescence intensity (Figure 3A). After 3 weeks, tumors were dissected and analyzed for weight and volume. Notably, the MZF1‐overexpressing group exhibited significantly larger tumor weights and volumes compared to the control group (Figure 3B–D). Immunoblotting and qPCR confirmed MZF1 overexpression in the xenografted tumors (Figure 3E,F). Moreover, immunohistochemical analysis revealed enhanced Ki67 expression in MZF1‐overexpressing tumors, further validating its role in promoting colon cancer cell proliferation in vivo (Figure 3G).

**FIGURE 3:**
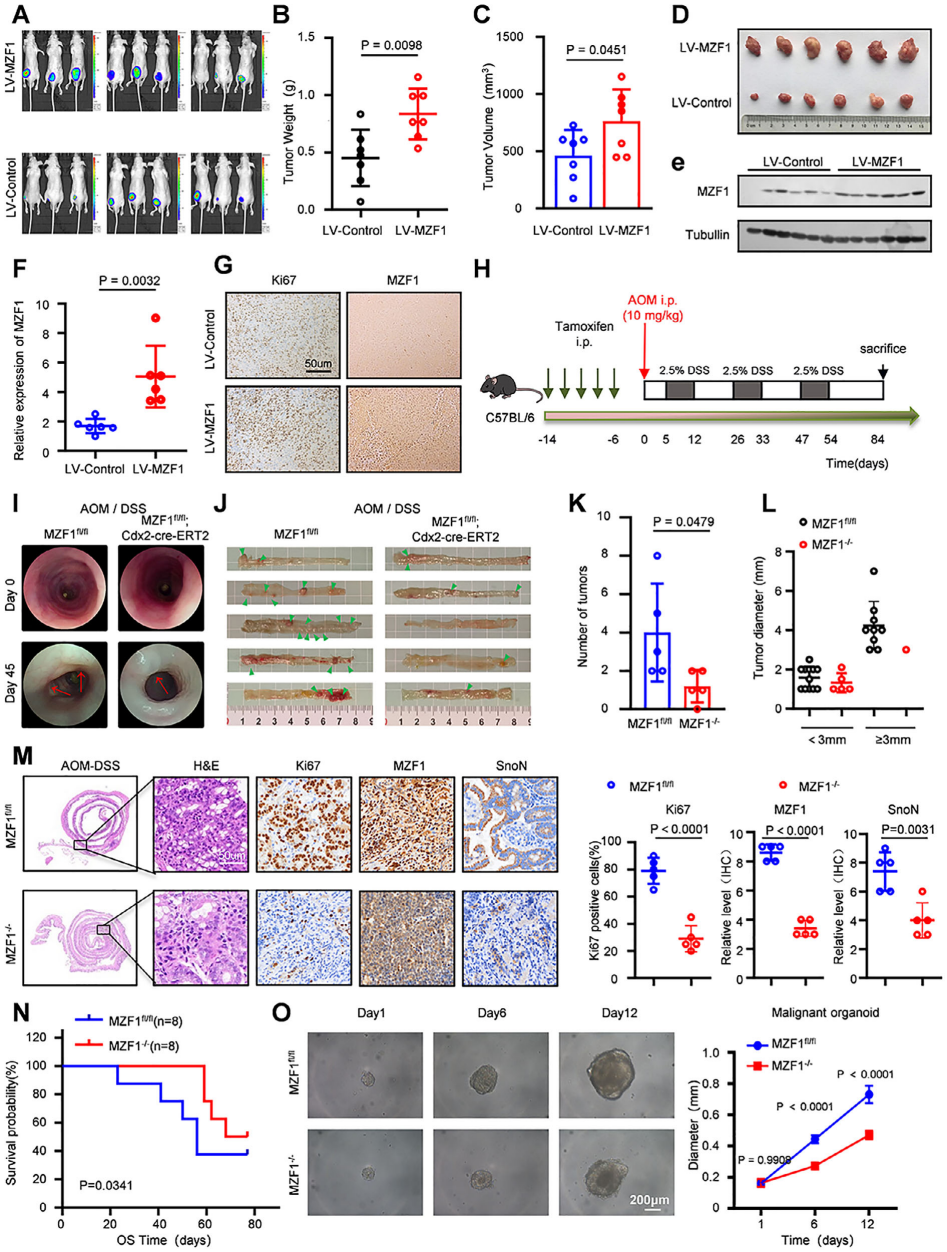
MZF1 is required for CRC tumor growth in vivo. (A) In vivo imaging of mice injected subcutaneously with HCT8 cells expressing luciferase and infected with control or MZF1 overexpression lentivirus. (*n* = 9 per group). (B,C) Tumor weight in grams (B) and volume in mm^3^ (C) of control or MZF1 overexpressing HCT8 xenograft tumors. (*n* = 7 per group). (D) Representative images of control or MZF1 overexpressing HCT8 xenograft tumors. (E,F) MZF1 protein measured by immunoblot (E) and *MZF1* mRNA measured by RT‐qPCR (F) levels of MZF1 in xenograft tumor tissues. (G) IHC analysis of MZF1 and Ki67 expression levels in xenograft tumor tissue. (H) Schematic representation of the AOM/DSS induced‐orthotopic CRC mouse model. (I) Representative images of colonoscopy surveillance 40 days after AOM injection. (J) Representative images (J) of colon from *MZF1^fl/fl^* and *MZF1^fl/fl^*;*Cdx2‐cre‐ERT2* mice after AOM/DSS model induction on harvesting. Green triangles indicate individual tumor nodules. (K,L) Tumor numbers (K) and tumor diameter (L) in AOM/DSS treated mice colons were determined. (M) Hematoxylin and eosin (H&E) staining and IHC staining of Ki67, MZF1, and SnoN expression levels in the AOM/DSS model mice. Representative images (left) and quantification (right). (N) Kaplan–Meier survival analysis of *MZF1^fl/fl^* and *MZF1^fl/fl^*;*Cdx2‐cre‐ERT2* mice after AOM/DSS‐induced orthotopic CRC model. (*n* = 8 per group). (O) Representative images (left) and quantification (right) of organoids formed from *MZF1^fl/fl^* and *MZF1^fl/fl^*;*Cdx2‐cre‐ERT2* mice orthotopic colon tumors (scale bars = 200 µm). All *p* values were calculated using two‐tailed, unpaired Student's *t*‐test.

To delineate the functional role of epithelial MZF1 in CRC development in vivo, we generated epithelial cell‐specific conditional MZF1‐deficient mice using the *Cre‐LoxP* system. By intercrossing a mouse line containing floxed alleles of *MZF1* (*MZF1^fl/fl^*) with another line expressing the *Cre* recombinase under control of the *Cdx2* promoter (*Cdx2‐Cre‐ERT2*) we generated intestinal epithelial cell‐specific *MZF1* knockout mice (*MZF1^fl/fl^*;*Cdx2‐cre‐ERT2*) and control mice (*MZF1^fl/fl^*). The genotypes of the resulting mice were verified using PCR analysis (Figure S3A,B). Next, to explore the oncogenic role of MZF1 in CRC tumorigenesis, we established a model of azoxymethane‐dextran sodium sulfate (AOM‐DSS)‐induced colorectal tumorigenesis in *MZF1^fl/fl^* and *MZF1^fl/fl^*;*Cdx2‐cre‐ERT2(MZF1^−/−^)* mice (Figure 3H). During the modeling process, we judged whether the modeling was successful by observing the hematochezia of mice with naked eyes and tumor formation in the colon through colonoscopy (Figure 3I and Figure S3C). There was no significant difference in body weight and colon length between the two groups (*P*>0.05) (Figure S3D,E). Remarkably, *MZF1^−/−^* mice had significantly fewer tumors, especially those with large size (≥3 mm), when compared with their *MZF1^fl/fl^* littermates (Figure 3I–K). Consistently, Ki67‐positive cells in the colorectal tissues from *MZF1^−/−^* mice were significantly decreased compared to *MZF1^fl/fl^* littermates (Figure 3M). Moreover, *MZF1^−/−^* mice had longer survival than *MZF1^fl/fl^* littermates mice (Figure 3N). Additionally, organoids derived from *MZF1^fl/fl^* mice grew more quickly and had a larger size in *ex vivo* culture, with larger diameters than organoids derived from *MZF1^−/−^* mice (Figure 3O). Taken together, these data suggest that overexpression of MZF1 can promote tumor growth in CRC, whereas MZF1 deletion attenuates colorectal tumorigenesis.

### 2.4 SnoN is a Critical Downstream Target of MZF1 Which Directly Binds to, and Activates, the *SnoN* Promoter

To elucidate the molecular mechanisms underlying MZF1‐mediated colon cancer cell proliferation, we performed RNA‐seq and CUT&Tag sequencing. First, RNA‐seq analysis of MZF1‐overexpressing colon cancer cells identified 4917 differentially expressed genes compared to controls (fold change >2 or <0.5, *p* < 0.05) (Table S9), which was visualized through a heatmap (Figure S4A) and volcano plot (Figure 4A). CUT&Tag sequencing revealed that MZF1 predominantly binds promoter regions across the genome, with peak distribution patterns shown in Figure 4B. Motif enrichment analysis identified canonical MZF1‐binding elements within these peaks (Figure 4C). Gene annotation further indicated MZF1 occupancy in the promoters of 9442 target genes (Tables S10 and S11). Intersection of RNA‐seq and CUT&Tag data identified 2418 direct MZF1‐regulated downstream genes (Figure S4B and Table S12), with GO functional annotation revealing enrichment in proliferation‐related pathways (Figure 4C).

**FIGURE 4:**
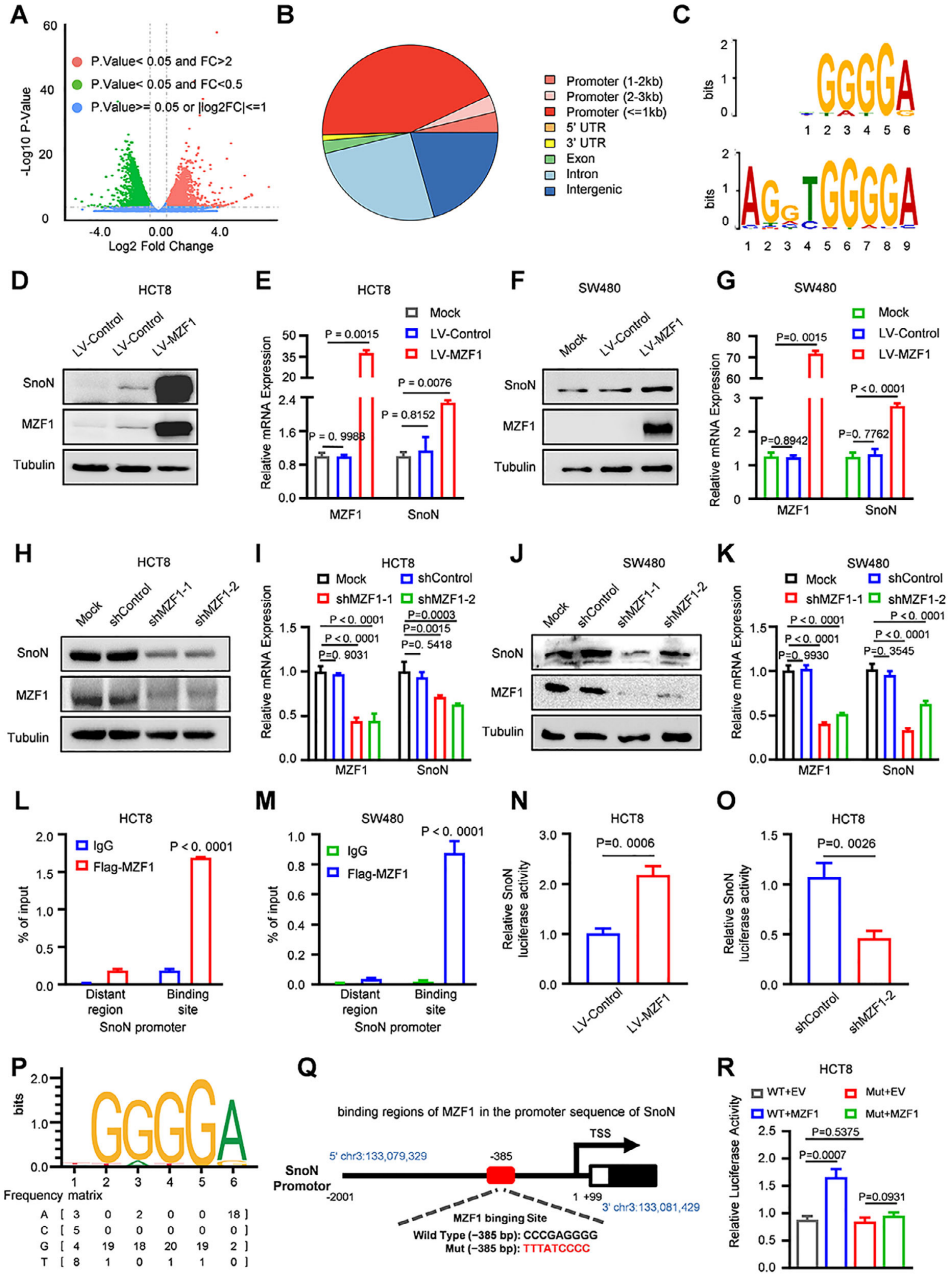
MZF1 transcriptionally activates SnoN in colon cancer. (A) Volcano plot of differentially expressed genes in SW480‐LVMZF1 vs. SW480‐LVControl cells determined by RNA sequencing. (B) The distribution ratio of peaks on gene structural elements obtained by CUT&Tag sequencing in HCT8 cells stably overexpressing MZF1‐Flag. (C) Motif enrichment analysis suggesting MZF1 binding elements in the peak sequences obtained by CUT&Tag sequencing. (D,E) Protein (D) and mRNA (E) levels of SnoN and MZF1 in HCT8 cells infected with control or MZF1 overexpression lentivirus and mock‐infected cells using immunoblotting (D) and RT‐qPCR (E). (F,G) Protein (F) and mRNA (G) levels of SnoN and MZF1 in SW480 cells infected with control or MZF1 overexpression lentivirus and mock‐infected cells using immunoblotting (D) and RT‐qPCR (E). (H,I) Protein (H) and mRNA (i) levels of MZF1 and SnoN in HCT8 cells infected with shControl or shMZF1‐expressing lentivirus and mock‐infected cells using immunoblotting (H) and RT‐qPCR (I). (J,K) Protein (J) and mRNA (k) levels of MZF1 and SnoN in SW480 cells infected with shControl or shMZF1‐expressing lentivirus and mock‐infected cells using immunoblotting (H) and RT‐qPCR (I). (L,M) ChIP‐qPCR experiments showing enrichment of MZF1 on the SnoN promoter region in HCT8 (M) and SW480 (N) cells. (N) Luciferase reporter assays of SnoN activity in HCT8 cells infected with control or MZF1 overexpression lentivirus (LV‐MZF1 or LV‐Control HCT8 cells). Luciferase activity was normalized against β‐gal activity. (O) Luciferase reporter assays of SnoN activity in HCT 8 cells infected with shControl or shMZF1‐expressing lentivirus. Luciferase activity was normalized against β‐gal activity. (P)​ Motif frequency matrix of the MZF1‐binding site identified in the SnoN promoter. The canonical GGGGA motif (bits score indicated) is highlighted. (Q) Schematic representation of the SnoN promoter region and the MZF1 binding site. The wild‐type (WT) sequence (−385 bp: CCCGAGGGG) and mutant (Mut) sequence (−385 bp: TTATCCCC) are indicated. (R) Luciferase reporter assays showing that MZF1 significantly activates the SnoNpromoter, whereas mutation of the −385 bp GGGG motif (Mut) abolishes this activation. Data are mean ± SD; *n* = 3.

Among the differentially expressed genes, proliferation‐associated genes *SnoN*, *CUL4B*, and *PHLDA3* emerged as notable candidates with well‐characterized oncogenic roles in colon cancer [14, 15, 16, 17]. RNA‐seq showed that *SnoN* and *CUL4B* were upregulated, while *PHLDA3* was downregulated in MZF1‐overexpressing cells compared to controls (Tables S9 and S12). However, subsequent qPCR and immunoblotting validation revealed that PHLDA3 was actually upregulated at both mRNA and protein levels, which was inconsistent with the RNA‐seq prediction; therefore, PHLDA3 was excluded from further functional investigation. Consistent with the RNA‐seq data for SnoN and CUL4B, qPCR and immunoblotting analyses confirmed that SnoN and CUL4B were significantly upregulated at both mRNA and protein levels in MZF1‐overexpressing HCT8 and SW480 cells (Figure 4D–G and Figure S5A–D), whereas depletion of MZF1 led to their downregulation (Figure 4H–K and Figure S5E–I). ChIP‐qPCR demonstrated that MZF1 binds specifically and significantly to the SnoN promoter region (*p* < 0.0001; Figure 4L,M and Figure S5J–M), whereas enrichment at the PHLDA3 and CUL4B promoters did not reach the predefined positive threshold. Luciferase reporter assays further supported this finding, showing enhanced SnoN promoter activity in HCT8 cells transfected with LV‐MZF1 compared with LV‐Control, whereas knockdown of MZF1 decreased SnoN activity (Figure 4N,O). To further define the critical cis‐regulatory element, motif analysis of the CUT&Tag peaks identified a canonical GGGGA motif at −385 bp relative to the SnoN transcription start site (TSS) (Figure 4P). We subsequently constructed a schematic of the SnoN promoter harboring either the wild‐type sequence or a mutant version in which the core GGGG motif was altered to TTTT (Figure 4Q). Luciferase reporter assays revealed that MZF1 overexpression significantly enhanced 4promoter activity in HCT8 cells, whereas this transcriptional activation was completely abolished by mutation of the GGGG motif (Figure 4R). Immunofluorescence staining in HCT8 cells revealed the positive correlation between MZF1 and SnoN expression (Figure S4D). In addition, immunohistochemical staining of CRC tissues from intestinal epithelial specific MZF1 knockout mice also revealed that, compared to the control group, the tamoxifen‐induced MZF1 knockout mice exhibited significantly lower levels of SnoN protein (Figure 3M). Collectively, these findings identify SnoN, but not PHLDA3or CUL4B, as a direct transcriptional target of MZF1. MZF1 directly binds to the −385 bp GGGG motif within the SnoN promoter to activate its transcription, thereby promoting colon cancer cell proliferation and tumorigenic progression.

### 2.5 MZF1 Transcriptional Activation of SnoN Regulates the TGF‐β Signaling Pathway to Promote Colon Cancer Cell Proliferation

We analyzed endogenous SnoN expression across multiple colon cancer cell lines and found that SnoN was significantly upregulated in colon cancer cells compared to normal colon epithelial cells (NCM460; Figure S6A). Subsequently, we constructed SnoN‐overexpressing cell lines (RKO, LOVO) and SnoN‐depleted cell lines (SW480, HCT8) (Figure S6B–E). Functional assays including CCK8, colony formation, and soft agar assays revealed that SnoN overexpression significantly enhanced colon cancer cell clonogenicity (Figure S6F–H) and proliferation capacity (Figure S6I–K), while SnoN depletion led to the opposite effects (Figure S6K–N). These results confirm a regulatory role for SnoN in colon cancer cell proliferation.

To determine whether the proliferative effects of MZF1 depend on SnoN upregulation, we depleted SnoN in MZF1‐overexpressing HCT8 and SW480 cells. Immunoblotting and qPCR confirmed effective MZF1 overexpression and SnoN depletion (Figure 5A–C). Subsequent CCK8 and colony formation assays revealed that depletion of SnoN reversed the proliferative and clonogenic promoting effects of MZF1 overexpression (Figure 5E–H). Conversely, restoring SnoN expression in MZF1‐depleted HCT8 or SW480 cells partially rescued the proliferation defects caused by MZF1 loss (Figure S7A–D). These findings establish a functional dependency of MZF1 proliferative activity on SnoN.

**FIGURE 5:**
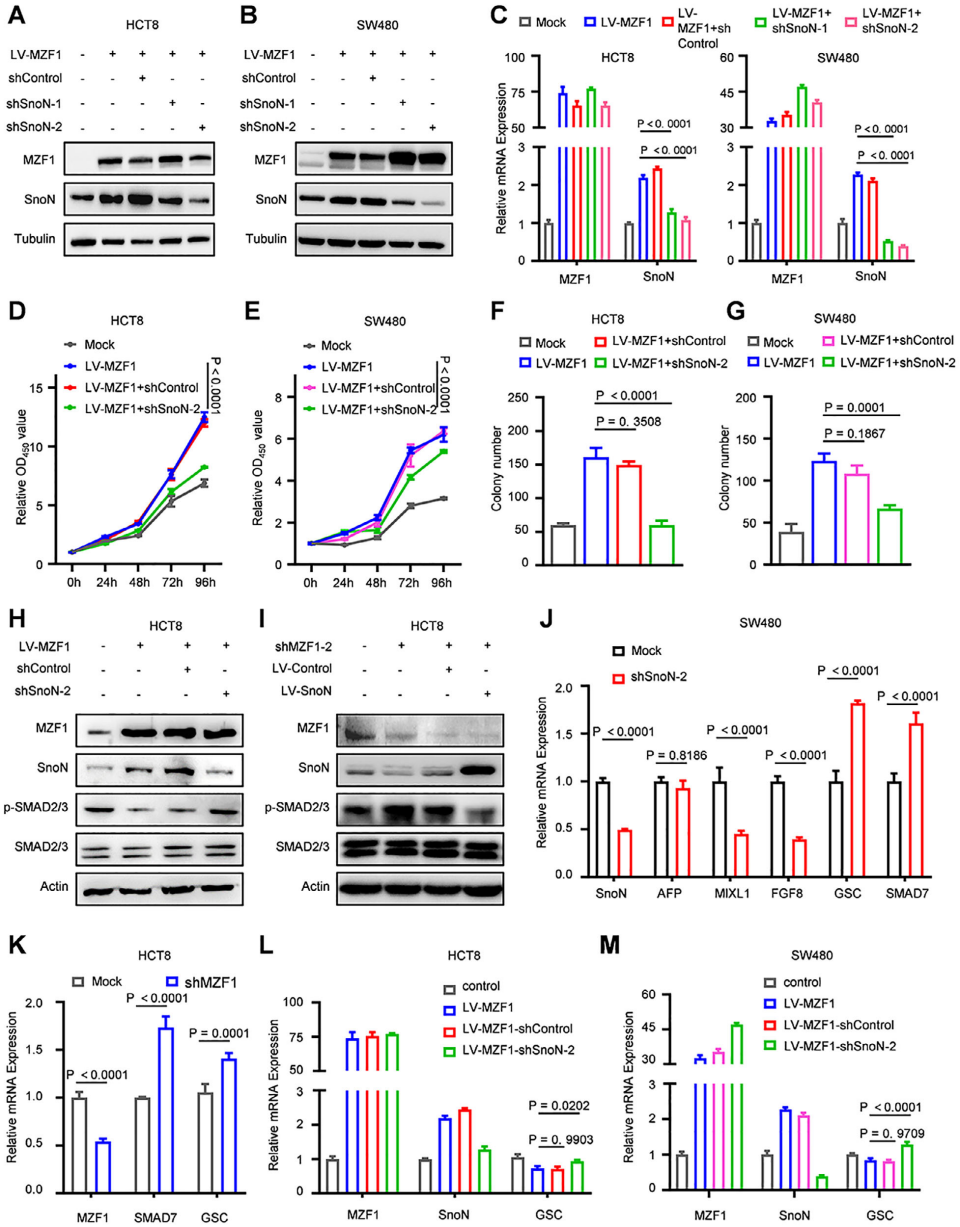
MZF1 transcriptionally activates SnoN and regulates TGF‐β signaling to promote colon cancer cell proliferation. (A) Immunoblotting of MZF1, SnoN, and tubulin in HCT8‐LV‐MZF1 cells infected with shControl or shSnoN expressing lentivirus or mock‐infected cells. (B) Immunoblotting of MZF1, SnoN, and tubulin in SW480‐LV‐MZF1 cells infected with shControl or shSnoN‐expressing lentivirus or mock‐infected cells. (C) RT‐qPCR of *MZF1*, *SnoN* in mock or HCT8‐LV‐MZF1 (left) and SW480‐LV‐MZF1 (right) cells infected with shControl or shSnoN‐expressing lentivirus. (D,E) CCK8 of mock or HCT8‐LV‐MZF1 (D) or SW480‐LV‐MZF1 (E) cells infected with shControl or shSnoN‐expressing lentivirus. (F,G) Quantification of colony formation assays of mock or HCT8‐LV‐MZF1 (F) or SW480‐LV‐MZF1 (G) cells infected with shControl or shSnoN‐expressing lentivirus. (H) Immunoblotting of MZF1, SnoN, SMAD2/3, p‐SMAD2/3, and actin in mock or HCT8‐LV‐MZF1 infected with shControl or shSnoN expressing lentivirus. (I) Immunoblotting of MZF1, SnoN, SMAD2/3, p‐SMAD2/3, and actin in HCT8‐ shMZF1 expressing cells infected with control or SnoN‐overexpressing lentivirus. (J) RT‐qPCR of known SnoN/SMAD downstream genes *SnoN, AFP, MIXL1, FGF8, GSC*, and *SMAD7* in SW480 cells either mock infected or infected with shSnoN‐expressing lentivirus. (K) RT‐qPCR of *MZF1, GSC*, and *SMAD7* in HCT8 cells either mock infected or infected with shMZF1‐expressing lentivirus. (L,M) RT‐qPCR of *MZF1, SnoN*, and *GSC* in HCT8‐LV‐MZF1 (L) or SW480‐LV‐MZF1 (M) cells either mock infected or infected with shSnoN‐expressing lentivirus. ANOVA was used for multiple comparisons; *p* < 0.05 is considered significant.

SnoN functions as a critical transcriptional co‐repressor of the SMAD protein family and plays essential roles in embryonic development and tissue homeostasis through TGF‐β1‐SMAD2/3 signaling regulation [14, 15, 16, 17]. Given its established role in promoting tumorigenesis via TGF‐β pathway antagonism across multiple cancers, we reasoned that MZF1 might regulate colon cancer progression through SnoN‐mediated TGF‐β‐SMAD2/3 inhibition. To assess this mechanism, we analyzed TGF‐β‐SMAD2/3 signaling in HCT8‐shMZF1‐LV‐SnoN, HCT8‐LVMZF1‐shSnoN cell lines by immunoblotting. MZF1 overexpression increased SnoN expression at both mRNA and protein levels without altering total SMAD2/3 levels, but significantly suppressed SMAD2/3 phosphorylation (p‐SMAD2/3). Notably, SnoN depletion partially rescued p‐SMAD2/3 suppression in MZF1‐overexpressing cells (Figure 5H). Furthermore, compared to shMZF1 and shMZF1‐LVControl cells, shMZF1‐LV‐SnoN cells revealed that LV‐SnoN restored SMAD2/3 phosphorylation to baseline levels (Figure 5I).

To further validate the functional impact of this regulation, we performed CAGA‐luciferase reporter assays. As shown in Figure S7J–K, MZF1 overexpression significantly inhibited TGF‐β‐induced CAGA‐luc activity, and this inhibition was partially reversed by SnoN knockdown, confirming that the MZF1/SnoN axis suppresses SMAD transcriptional output.

Genes including *SMAD7*, *GSC*, *FGF8*, *MIXL1*, and *AFP* as targets of the SnoN/SMAD complex [14, 15, 16, 17]. We performed qPCR analysis of these downstream genes in colon cancer cells and observed that *GSC* and *SMAD7* expression was upregulated upon SnoN and MZF1 depletion (Figure 5J,K). Moreover, SnoN depletion rescued *GSC* mRNA downregulation caused by MZF1 overexpression (Figure 5L,M). Collectively, these data suggest that SnoN acts as a co‐factor in MZF1 repression of TGF‐β1/SMAD2/3 signaling, thereby promoting colon cancer cell proliferation through coordinated regulation of SnoN expression and TGF‐β pathway activity.

### 2.6 Inhibition of MZF1 by 10Panx Acetate Suppresses Colon Cancer Cell Proliferation and Tumorigenesis Both In Vitro and In Vivo

To identify small‐molecule inhibitors targeting MZF1, we employed AlphaFold 3 (AF3) to predict the 3D structure of MZF1 (NCBI; Figure 6B).

**FIGURE 6:**
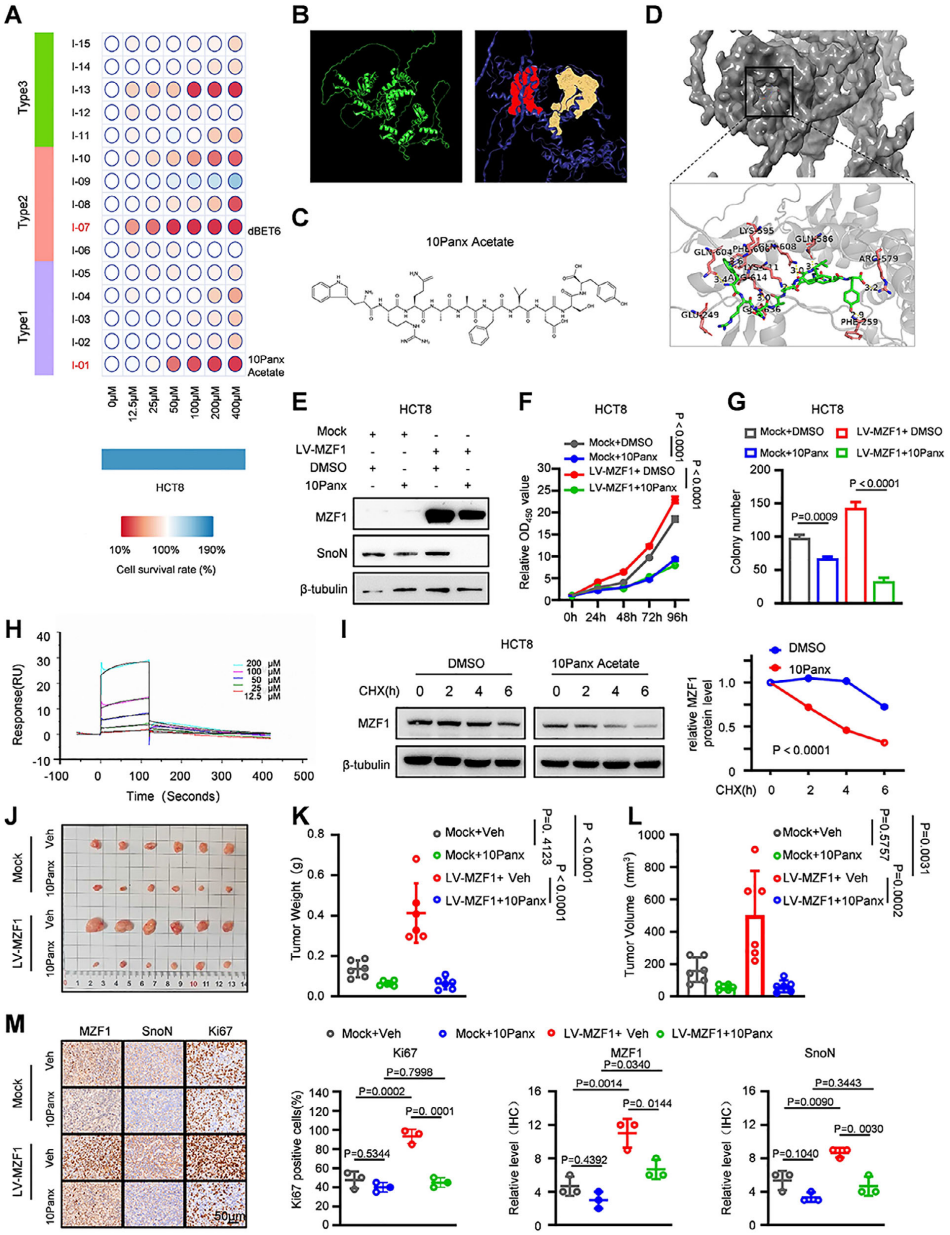
MZF1 inhibitor 10Panx Acetate suppresses colon cancer cell proliferation in vitro and colon cancer tumorigenesis in vivo. (A) Heatmap of HCT8 cell survival rate for 15 compounds using cell viability assay. (B) Schematic diagram of MZF1 3D structure (left) and MZF1 internal cavity (right). (C) Chemical structure of 10Panx Acetate polypeptide. (D) Predicted interaction diagram of MZF1 with 10Panx Acetate. (E) Immunoblotting for MZF1, SnoN, and tubulin in HCT8 cells with or without MZF1 overexpression treated in the presence or absence of 10Panx Acetate. (F) CCK8 assays in HCT8 cells with or without MZF1 overexpression treated in the presence or absence of 10Panx Acetate. (G) Quantification of colony formation assays of HCT8 cells with or without MZF1 overexpression treated in the presence or absence of 10Panx Acetate. (H) SPR assay to detect the binding between 10Panx Acetate and MZF1 protein. (I) Representative immunoblots (left) and quantification (right) of HCT8 cells treated for the indicated times with cycloheximide (CHX) (100 µg/mL) in the presence or absence of 10Panx Acetate. (J–L) Images of tumors (j), tumor weights (k) and tumor volumes (l) of xenografts either mock or overexpressing MZF1 (LV‐MZF1) cells treated with 10Panx Acetate (10 mg/kg) or saline. (*n* = 6 per group). (M) IHC staining and quantification (*n* = 4 per group) of MZF1, SnoN, and Ki67, levels in indicated xenograft tumors. Unless indicated otherwise, all assays were performed in triplicate. ANOVA was used for multiple comparisons; *p* < 0.05 is considered significant.

Guided by the predicted structure, we selected two potential ligand‐binding pockets and one putative active site for virtual screening of a commercial compound library, which initially yielded 15 candidate compounds (Table S13), including five peptides, five synthetic compounds, and five natural products. Primary screening for anti‐proliferative activity in HCT8 and SW480 cell lines (Figure 6A and Figure S8A) identified I‐01 (10Panx Acetate) and I‐07 (dBET6) as the most promising hits (Figure 6C,D and Figure S8B,C). Treating MZF1‐overexpressing HCT8 cells with 10Panx Acetate led to a significant reduction in SnoN mRNA levels without altering MZF1 transcript abundance (Figure S9A). Consistent with this, immunoblotting confirmed that 10Panx Acetate downregulated both MZF1 and SnoN protein levels in a time‐dependent manner, with more pronounced effects in MZF1‐overexpressing HCT8 and SW480 cells (Figure 6E and Figure S8D). Cycloheximide chase assays further revealed that 10Panx Acetate accelerated MZF1 protein degradation compared to the DMSO control (Figure S9B), suggesting that the reduction in MZF1 protein occurs at the post‐translational level. To functionally validate the anti‐tumor efficacy of 10Panx Acetate, we performed CCK8 proliferation and colony formation assays in mock or MZF1‐stably transfected HCT8 and SW480 cells. Treatment with 10Panx Acetate resulted in a more robust suppression of cell proliferation and clonogenic survival in MZF1‐overexpressing cells compared to their mock counterparts (Figure 6F,G and Figure S8E–H). To determine whether this anti‐proliferative effect was dependent on MZF1, we performed loss‐of‐function assays using shRNA‐mediated MZF1 knockdown. Notably, knockdown of MZF1 markedly attenuated 10Panx Acetate‐mediated suppression of proliferation and colony formation in HCT8 cells (Figure S8I–K), establishing that 10Panx Acetate functions in an MZF1‐dependent manner. In parallel, we evaluated the bromodomain inhibitor dBET6. While truncated peptide sequences derived from dBET6 (Sequence 1: D{D‐SER}{D‐TYR}; Sequence 2: WRQ; or their combination) failed to inhibit colorectal cancer cell viability (Figure S9C–E), the full‐length dBET6 effectively suppressed proliferation and colony formation in both HCT8 and SW480 cells, with enhanced efficacy observed in MZF1‐overexpressing contexts (Figure S8L–Q). However, unlike 10Panx Acetate, dBET6 did not reduce MZF1 protein levels; instead, Western blot analysis revealed a slight compensatory upregulation of MZF1 accompanied by a significant reduction in SnoN expression (Figure S9F). RT‐qPCR assays further demonstrated that dBET6 downregulated canonical BET family targets, including c‐MYC, BRD4, HGF, ISG15, and LAMB3, in MZF1‐overexpressing HCT8 cells (Figure S9F), indicating that its anti‐proliferative effects are primarily mediated through broad bromodomain inhibition rather than direct targeting of MZF1. Surface plasmon resonance (SPR) analysis confirmed that 10Panx Acetate directly binds to the MZF1 protein with a dissociation constant (KD) of 65.8 µm (Figure 6H), supporting its role as a specific MZF1 inhibitor. Finally, we evaluated the therapeutic potential of 10Panx Acetate in a subcutaneous xenograft mouse model. Treatment with 10Panx Acetate (10 mg/kg) significantly reduced tumor volume and growth rates in mice bearing MZF1‐overexpressing xenografts compared to vehicle‐treated controls (Figure 6J–L and Figure S9G), without causing overt alterations in body weight (Figure S9G). Histopathological examination of heart, liver, spleen, lung, kidney, and brain tissues by H&E staining revealed no detectable organ toxicity in 10Panx Acetate‐treated mice (Figure S9H). Immunohistochemical analysis of the excised tumors further confirmed that 10Panx Acetate treatment decreased the expression of MZF1 and SnoN proteins and reduced the proportion of Ki67‐positive proliferating cells (Figure 6M). Collectively, these findings establish 10Panx Acetate as a direct inhibitor of the MZF1‐SnoN signaling axis with potent anti‐tumor efficacy and a favorable safety profile in preclinical models of colorectal cancer.

### 2.7 SnoN is Highly Expressed in Clinical Samples and Associates With Poor Prognosis

Analysis of TCGA and GTEx databases revealed that *SnoN* mRNA levels were significantly higher in colorectal adenocarcinoma (READ) and colon adenocarcinoma (COAD) tissues compared to matched normal counterparts (Figure 7A,B and Figure S10A,B). To validate this observation, immunoblotting analysis of fresh colorectal cancer and adjacent normal tissues demonstrated that both MZF1 and SnoN were predominantly overexpressed in tumor tissues relative to normal tissues (Figure S10C). Immunofluorescence staining of fresh tumor samples further confirmed co‐expression of MZF1 and SnoN (Figure 7E). Immunohistochemical analysis of 76 CRC patients revealed that MZF1 and Ki67 were mainly localized in the nucleus, while SnoN was abundantly expressed in both cytoplasmic and nuclear compartments. Notably, MZF1, SnoN, and Ki67 exhibited significantly higher expression levels in tumor tissues compared to normal margins (Figure 7C,D; *p* < 0.05). Moreover, a positive correlation was observed between MZF1 and SnoN protein expression levels in tumor tissues (Figure 7F; Spearman's r = 0.3505, *p* < 0.05).

**FIGURE 7:**
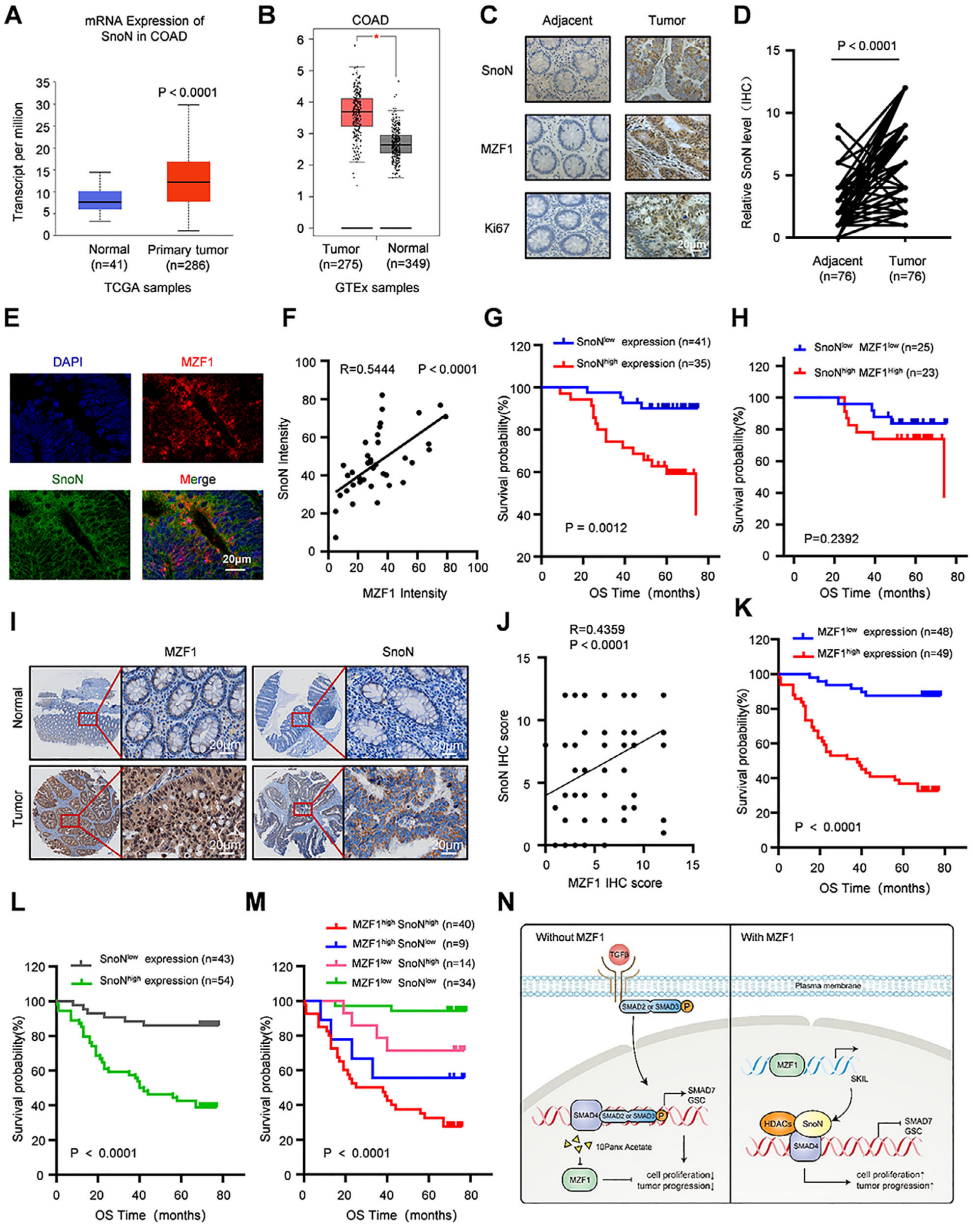
MZF1 and SnoN are both overexpressed in colon cancer tissues, and their co‐overexpression correlates with poor prognosis. (A) TCGA datasets were analyzed to examine the expression of *SnoN* in COAD samples (*n* = 286) and normal tissues (*n* = 41). (B) GTEx datasets were analyzed to examine the expression of *SnoN* in COAD samples (*n* = 349) and normal tissues (*n* = 275). (C,D) Representative IHC images of SnoN, MZF1, and Ki67 staining expression levels in tumor tissue and normal tissue of CRC patients (C). Quantification of SnoN expression levels in tumor tissue and corresponding normal tissue (*n* = 76). (E) Immunofluorescence analysis of tumor tissue samples from CRC patients showed co‐expression of MZF1 (red) and SnoN (green) in CRC tumors. (F) Scatter plot showing the positive correlation between MZF1 and SnoN IHC intensity in CRC tissues (R = 0.5444, *p* < 0.0001). (G) Kaplan–Meier survival analysis of tumors with SnoN^low^ (n‐41) and SnoN^high^ (*n* = 35) expression level for patient OS prognosis. (H) Kaplan–Meier survival analysis of tumors with MZF1^low^SnoN^low^ (*n* = 25) and MZF1^high^SnoN^high^ (*n* = 23) expression level for patient OS prognosis. (I) IHC analysis of MZF1 and SnoN expression in tumor tissues (*n* = 83) and adjacent normal tissues (*n* = 97) in SOC cohort. Scale bars: 50 µm. (J) Spearman correlation analysis of MZF1 and SnoN IHC scores in tumor tissues (*n* = 97). (K–M) Kaplan–Meier survival analysis of low vs. high expression of MZF1 (K), SnoN (L), or MZF1 and SnoN (M) expression level for patient OS prognosis in SOC cohort. (N) Schematic representations of the role of MZF1/SnoN axis in driving cell proliferation and tumor progression in CRC via the TGF‐β/SMAD signaling pathway.

To explore a role for SnoN in CRC progression and prognosis, we stratified the 76 patients based on SnoN expression levels. Patients with SnoN expression above the median (high group, *n* = 35) were compared to those below the median (low group, *n* = 41) (Figure S10D). Kaplan‐Meier survival analysis demonstrated that high SnoN expression was associated with shorter overall survival (OS; *p* = 0.0012) and metastasis‐free survival (MFS; *p* = 0.0489) compared to patients with low SnoN expression (Figure 7G and Figure S10F). However, no significant survival difference was observed when comparing patients with concurrent high/low MZF1 and SnoN expression groups (Figure 7H). Given the limited sample size of our cohort, we expanded our analysis using immunohistochemistry on CRC microarrays (SOC and AFC cohorts). Both MZF1 and SnoN exhibited significantly higher immunohistochemical scores in tumor tissues vs. normal margins (Figure 7I and Figure S10G–K). Spearman's correlation confirmed a strong positive association between MZF1 and SnoN scores in cancer tissues (Figure 7J and Figure S10L; r = 0.623, *p* < 0.001). Kaplan–Meier analysis revealed that high MZF1 (Figure 7K and Figure S10M) and high SnoN (Figure 7L and Figure S10N) immunohistochemical scores independently predicted shorter OS. Therefore, we then categorized patients into four groups based on MZF1/SnoN immunohistochemical scores: MZF1^low^/SnoN^low^, MZF1^low^/SnoN^high^, MZF1^high^/SnoN^low^, and MZF1^high^/SnoN^high^. Survival analysis showed that the MZF1^high^/SnoN^high^ group had the poorest OS (Figure 7M and Figure S10O). Univariate and multivariate Cox regression analyses identified high MZF1 and SnoN scores as independent risk factors for mortality in SOC cohort (HR = 15.143, 95% CI: 3.538–64.806; *p* = 0.000) or AFC cohort (HR = 19.918, 95% CI: 2.597–152.751; *p* = 0.004) (Table S14). Collectively, these data establish that MZF1 and SnoN are co‐overexpressed in CRC and their combined overexpression serves as a prognostic biomarker for adverse outcomes.

## 3 Discussion

Our understanding of TFs in both normal cells and tissues as well as in cancer has become more advanced. As a class of proteins capable of binding to specific DNA sequences to regulate gene transcription, TFs play a crucial role in various biological processes such as cell growth, differentiation, and apoptosis [18, 19]. Aberrant expression or altered activity of TFs is often closely associated with the initiation, progression, and metastasis of tumors [8, 20]. Multiple studies have shown that tumor proliferation is often accompanied by abnormal expression of TFs, which subsequently leads to aberrant expression of oncogenes and tumor suppressor genes, promoting tumor progression [21, 22]. Therefore, by searching the GEO database, we collected gene expression profiles from 1215 CRC and adjacent normal tissue samples. Using bioinformatics techniques, we analyzed the differentially expressed genes in tumor and adjacent normal tissues. Subsequently, through transcriptional co‐regulation analysis of the differentially expressed genes in CRC tissues, we found MZF1 is a prominent transcription factor regulating gene expression in CRC. MZF1 belongs to the zinc finger transcription factor subfamily. Regarding the role of MZF1 in tumorigenesis and progression, previous studies have shown that its gene copy number is significantly altered in tumors such as breast cancer [23], lung cancer [24], bladder cancer [25], glioma [26], and colon cancer [27]. It also plays a role in promoting tumor progression through regulating apoptosis, cell migration and invasion, cell proliferation, and stem cell properties [28, 29, 30]. However, there is relatively little known regarding MZF1 in CRC. MZF1 in CRC cells can induce tumor cell proliferation, migration, and invasion by binding to the *Axl* promoter to enhance *Axl* mRNA and protein expression [31]. Another study revealed that MZF1 enhances tumorigenic capacity in CRC by transcriptionally activating the regulatory subunit p55PIK of IA‐PI3K, thereby activating the PI3K/Akt pathway [32]. Conversely, it has been shown that sulindac sulfide promotes colon cancer cell apoptosis by upregulating *MZF1* to induce the expression of *DR5* in TRAIL [33]. Therefore, whether MZF1 plays a pro‐oncogenic or tumor‐suppressive role in CRC remains uncertain, and the mechanisms by which it exerts its effects require further investigation. In this study, through mining the TCGA database and immunohistochemical (IHC) analysis of clinical colon cancer samples, we found that MZF1 is highly expressed in CRC tissues compared to normal tissues. Patients with high MZF1 expression have shorter overall survival (OS), disease‐free survival (DFS), and metastasis‐free survival (MFS) than those with low MZF1 expression. This further suggests that MZF1 may be an important transcription factor regulating the initiation and progression of CRC, and MZF1 expression in CRC is a potentially important target, with its individual effect being sufficient to influence patient survival.

To further clarify the biological functions of MZF1 in CRC, we assessed the effects of MZF1 at the cellular level. Using both overexpression or depletion of MZF1 levels, we observed the effect of MZF1 on the proliferation ability of CRC cells in vitro and in vivo. Our results showed that overexpression of MZF1 significantly enhanced the proliferation ability of CRC cells, while depletion of MZF1 significantly inhibited CRC proliferation. The use of inducible intestinal epithelial‐specific MZF1 knockout mice in an AOM/DSS chemically induced orthotopic colon cancer model further confirmed that knockout of MZF1 inhibits the development of colon cancer. It is worth noting that although clinical sample analysis revealed that MZF1 levels correlate with lymphatic metastasis and reduced MFS, our preliminary in vitro assays (including Transwell migration and wound healing) failed to detect a significant effect of MZF1 on CRC cell motility.​ This discrepancy suggests that the association between MZF1 and metastatic outcomes in patients may not be attributable to a direct pro‐metastatic function of MZF1 (e.g., inducing EMT or invasion), but rather reflects the clinical sequelae of accelerated primary tumor growth. In the context of early to mid‐stage CRC, where TGF‐β signaling predominantly exerts tumor‐suppressive effects, MZF1‐mediated SnoN upregulation and subsequent TGF‐β/SMAD inhibition primarily drive uncontrolled cellular proliferation. A larger, rapidly proliferating primary tumor inherently increases the likelihood of local invasion and provides a greater reservoir of circulating tumor cells, thereby indirectly contributing to shorter PFS and MFS. Therefore, we focused our mechanistic investigations on the role of MZF1 in promoting CRC cell proliferation and deciphering the downstream TGF‐β/SnoN axis.

Transcription factors aberrantly expressed in tumor cells usually bind to transcription factor binding sites upstream of target genes to control gene expression of factors that may contribute to regulating a series of malignant biological behaviors such as cell proliferation and metastasis [34, 35]. To explore the mechanisms by which MZF1 promotes colon cancer proliferation, we took the intersection of RNA sequencing and CUT‐tag sequencing results regulated by MZF1 and searched for downstream proliferation‐related target genes transcriptionally regulated by MZF1 among the intersecting genes. We found the proliferation‐related genes *SnoN*, *PHLDA3*, and *CUL4B* were significantly affected by MZF1 and have been reported to be studied in colon cancer. CUL4B is a member of the Cullin protein family and participates in the formation of the Cullin‐Ring ubiquitin ligase complex (CRLs). Studies on CUL4B in CRC have shown that it promotes colorectal cancer progression. For example, CUL4B mediates the degradation of the histone demethylase UTX to promote CRC progression [14, 15, 16, 17], and CUL4B promotes the proliferation and invasion of CRC cells by inhibiting the p53 signaling pathway [14, 15, 16, 17]. Pleckstrin homology domain family A member 3 (PHLDA3) is a target gene of the tumor suppressor p53 and encodes an inhibitor of Akt, exerting tumor‐suppressive effects in various cancers such as pancreatic neuroendocrine tumors (PanNETs) [14, 15, 16, 17]. SnoN is a key transcriptional co‐factor of the SMAD transcription factors and is encoded by the human SKI‐like (SKIL) gene [14, 15, 16, 17]. Ski/SnoN disrupts the formation of the R‐SMAD/SMAD4 complex and inhibits the binding of SMAD to the p300/CBP co‐activator, thereby interfering with the biological effects of the TGF‐β/SMAD signaling pathway. Ski/SnoN recruits multiple co‐repressors and histone deacetylases to inhibit gene transcription [14, 15, 16, 17]. Studies have shown that SnoN is upregulated in various tumors such as leukemia [36], breast cancer [37], and gastrointestinal tumors [38]. In addition, SnoN can promote the proliferation of tumor cells such as pancreatic and colon cancer by directly binding to P‐SMAD2/3 and SMAD4, blocking the formation of the transcriptional complex, and mediating the negative regulation of TGF‐β signaling [39, 40]. In this study, we show that the transcription factor MZF1 directly binds to, and transcriptionally activates, the downstream target gene *SnoN*. The MZF1/SnoN axis can affect the expression of TGF‐β downstream target genes *SMAD7* and *GSC*, and we found that MZF1 promotes the proliferation of colon cancer cells through SnoN, suggesting that the MZF1/SnoN axis may promote CRC proliferation by inhibiting the TGF‐β signaling pathway.

As an oncogenic gene, inhibiting the activity of MZF1 may provide new opportunities for anti‐cancer treatment. To identify small‐molecule inhibitors of MZF1, we screened a database of approved drugs and identified 10Panx Acetate as a potential binder of MZF1. In previous studies, 10Panx Acetate, as a mimetic inhibitory peptide, binds to and reversibly inhibits Panx1 and is used in the management of neuroinflammation and pain [41, 42]. In addition, it may have certain application value in the treatment of infectious diseases [43]. However, its role as a potential anti‐cancer agent has not been fully explored. Our experimental results reveal that 10Panx Acetate is able to inhibit the proliferation ability of colon cancer cells with enhanced activity in cells overexpressing MZF1. Using a subcutaneous xenograft model we further verified that 10Panx Acetate targets and inhibits the MZF1/SnoN axis to suppress tumor progression in vivo, exerting potent therapeutic efficacy without toxicity to various organs. In addition, SPR analysis confirm that 10Panx Acetate can directly bind to the MZF1 protein. Mechanistically, 10Panx Acetate may inhibit the function of MZF1 by accelerating its protein degradation. In summary, 10Panx Acetate, as a targeted inhibitor of MZF1, may serve as a novel therapeutic drug for colon cancer. Notably, while virtual screening identified dBET6 as a candidate inhibitor, our comprehensive functional and mechanistic analyses revealed fundamental differences that guided our prioritization of 10Panx Acetate. Although dBET6 suppressed the proliferation of MZF1‐overexpressing CRC cells, it functioned as a pan‐bromodomain inhibitor rather than a specific MZF1 antagonist. Mechanistically, dBET6 downregulated canonical BET targets, including BRD4 and c‐MYC, and reduced SnoN expression without destabilizing the MZF1 protein; indeed, we observed a compensatory upregulation of MZF1 following dBET6 treatment. In stark contrast, 10Panx Acetate directly engaged MZF1, as confirmed by SPR analysis (KD = 65.8 µm), and facilitated its proteasomal degradation, leading to the specific abrogation of the MZF1‐SnoN axis. This direct mode of action translated into superior anti‐tumor efficacy in vivo, where 10Panx Acetate significantly impeded the growth of MZF1‐overexpressing xenografts without discernible systemic toxicity. Therefore, while dBET6 served as a valuable tool to validate the therapeutic relevance of targeting MZF1‐driven proliferation, 10Panx Acetate represents a far more precise chemical probe. Our findings collectively underscore that 10Panx Acetate is a novel and selective MZF1 degrader, offering a robust pharmacological strategy to intercept the MZF1‐SnoN signaling cascade in colorectal cancer. While 10Panx Acetate effectively suppressed tumor growth and the MZF1‐SnoN axis in subcutaneous xenografts without overt toxicity, we acknowledge that subcutaneous models have limitations in recapitulating the clinical tumor microenvironment. Future studies employing orthotopic or patient‐derived xenograft (PDX) models are warranted to validate the therapeutic efficacy of 10Panx Acetate in a more clinically relevant context. Additionally, comprehensive pharmacokinetic profiling and tissue distribution analysis will be essential to determine the optimal dosing regimen and confirm the dose‐dependent inhibition of the MZF1‐SnoN axis in vivo.

To verify the expression characteristics and prognostic value of MZF1 and SnoN in CRC, we used patient samples collected from our hospital and two human CRC tissue microarrays for prognostic evaluation. We found that MZF1 protein levels are significantly elevated in human CRC samples. Furthermore, MZF1 overexpression is closely associated with poor prognosis in CRC patients. In addition, high expression of SnoN in CRC tissues is predictive of poor patient prognosis. More importantly, patients with high levels of both MZF1 and SnoN had a worse prognosis than other groups. These results indicate that the protein levels of MZF1 and SnoN in tumor tissues can serve as reliable prognostic evaluation indicators.

In conclusion, we identified MZF1 as a transcription factor that plays a critical role in promoting CRC cell proliferation and tumor progression. MZF1 targets the SMAD transcriptional co‐factor SnoN and regulates the TGF‐β signaling pathway, thereby promoting CRC. The MZF1 inhibitor 10Panx Acetate showed efficacy in both in vitro and in vivo models of colorectal cancer, highlighting that targeting MZF1, through 10Panx Acetate, may serve as a potential target for CRC treatment.

## 4 Materials and Methods

### 4.1 Cell Culture and Reagents

NCM460(RRID: CVCL_0460), HCT8(RRID: CVCL_2478), HCT15(RRID: CVCL_0292), HCT116(RRID: CVCL_0291), SW480(RRID: CVCL_0546), RKO(RRID: CVCL_0504), Lovo(RRID: CVCL_0399), and SW620(RRID: CVCL_0547) cell lines were procured from the cell bank of the Shanghai Institute of Biochemistry and Cell Biology (Shanghai, China). NCM460, HCT8, HCT15, and Lovo were cultured in RPMI 1640 medium (Gibco, USA) supplemented with 10% fetal bovine serum (Excell, USA) and 100X Penicillin‐Streptomycin Solution (NCM, China). HCT116 cells were maintained in McCoy's 5A medium (Gibco, USA) with 10% fetal bovine serum (Excell, USA) and 100X Penicillin‐Streptomycin Solution (NCM, China). RKO cells were cultured in MEM medium (Gibco, USA) supplemented with 10% fetal bovine serum (Excell, USA) and 100X Penicillin‐Streptomycin Solution (NCM, China). All cell lines were incubated at 37°C in a 5% CO_2_ atmosphere and tested negative for mycoplasma contamination.

### 4.2 Patients and Samples

A total of 96 colon cancer tissue samples were collected immediately following surgical resection at Jinling Hospital, Nanjing University. The selection criteria for these samples included a pathological diagnosis of primary colorectal cancer, complete clinical pathological data, and no restriction on the gender of the participants. Informed consent was obtained from those patients participating in this study, and the study received approval from the Ethical Review Committee of Jinling Hospital, Nanjing University. Additionally, human colorectal cancer tissue microarray (HColA‐180Su23, SOC cohort) was purchased from Shanghai Outdo Biotech and the CRC tissue microarray (AF‐CocSur2201, AFC cohort) was purchased from AiFang Biological. All enrolled participants finished the study, i.e., there was no attrition.

### 4.3 Soft Agar Assays

Using six‐well plates, solid consisting of two layers were prepared. The first consisted of 2.5% melting Nobel agar was mixed with RPMI 1640 to make 0.3% and 0.6% agar at 50°C. The 0.6% agar were added to the bottom layer and the 0.3% agar was suspended with 1 × 10^4^ cells were added to the top layer. Then 500 µL complete RPMI 1640 medium was added to maintain top layer moisture. After 2–3 weeks, the cells were stained with iodonitrotetrazolium chloride (1 µg/mL) (AMRESCO, #0294‐1G) for colony visualization and counting. Three independent experiments were performed to generate mean and standard deviation (S.D.).

### 4.4 Immunohistochemistry

Tissue sections were deparaffinized in xylene and subjected to a series of graded ethanol concentrations. Following treatment with EDTA antigen retrieval solution and goat serum, the slides were incubated overnight at 4°C with primary antibodies, including MZF1 (Thermo Fisher, #PA5‐106478, 1:300, RRID: AB_2854147), SnoN (Thermo Fisher, #MA5‐26386 1:150, RRID: AB_2723661), and Ki67 (Servicebio, # GB111499, 1:500, RRID: AB_ 3083641). Subsequently, the tissue sections were incubated with the appropriate secondary antibodies and treated with DAB, followed by hematoxylin staining for subsequent microscopic observation.

For immunohistochemical scoring, four random high‐magnification fields of view were selected and assessed in a double‐blind manner by two experienced pathologists, based on the percentage of positive cells and the intensity of positive cell staining. The intensity of staining was categorized into four grades: 0 for no staining, 1 for weak staining, 2 for moderate staining, and 3 for strong staining. Concurrently, the percentage of positive cells was classified into five grades: 0 for no stained cells, 1 for <25%, 2 for 26%–50%, 3 for 51%–74%, and 4 for ≥75%. The immunohistochemical score for each sample was calculated using the formula: score = intensity of staining × proportion of positive cells stained.

### 4.5 Animal Experiments and Treatments

All animal experiments adhered to the Guide for the Care and Use of Laboratory Animals and received approval from the Animal Ethics Committee of Jinlin Hospital, Nanjing University. Mice were randomly grouped by age and weight prior to tumor cell inoculation. To ensure all treatment groups started with similar tumor burdens, tumor sizes were normalized before treatment commencement. All animals were housed in a standardized, pathogen‐free environment and fed a consistent mouse diet. Tumor dimensions were recorded using calipers, and the volume was calculated as: 0.5 × length × width^2^ (in cm^3^). Mice showing any sign of distress including tumors exceeding 2 cm, ulcers surpassing 5 mm, weight reduction of  ≥20%, or exhibiting a hunchback posture were humanely euthanized.

Subcutaneous mouse models to observe the effects of treatments including the MZF1 inhibitor 10Panx Acetate and stable MZF1 overexpression, both alone or in combination. HCT8‐LVControl‐Luc or HCT8‐LVMZF1‐Luc cells were prepared by resuspending 1 × 10^6^ cells in 100 µL of a 1:1 PBS:Matrigel mixture and injected subcutaneously into the left anterior superior iliac spine of 6‐week‐old male BALB/c nude mice(RRID: CVCL_0584). Three weeks after injection, the mice were euthanized in accordance with ethical guidelines, and the tumors were excised, measured for weight and volume, and subjected to hematoxylin and eosin (HE) staining or immunohistochemistry. For the administration of the MZF1 inhibitor 10Panx Acetate, HCT8 LVControl or HCT8‐LVMZF1 cells were selected and prepared for subcutaneous injection into nude mice. After 2 weeks, they were treated with 10Panx Acetate (TargetMol, #T21839L) at a dose of 10 mg/kg or saline via intraperitoneal injection (i.p.) every 3 days for 2 weeks.

The AOM/DSS‐induced colorectal cancer model: We constructed intestinal epithelial‐specific conditional *MZF1* knockout mice based on the *Cre‐LoxP* system. Genotyping for *Mzf1^fl/fl^* used the primers TAGAGTGGACTCTGGGATTCCAG and ACCCTGAGAGAAACTTGGAACAG with the expected product size of 233 bp for the wild‐type allele and 314 bp for the floxed allele and primers ATATGGAGCAAGCGAACGAGAG and CCAACTGACCTTGGGCAAGAACAT with the expected product size of 0 bp for the wild‐type allele and 337 bp for the floxed allele. Genotyping for *Cdx2‐cre‐ERT2* used the primers AGGTGTAGAGAAGGCACTTAGC CTAATCGCCATCTTCCAGCAGG with the expected product size of 443 bp for positive mice.

To induce cre recombinase activity, tamoxifen (240 µL of 20 mg/mL solution) was injected 5 times on alternate days into mice with *Cdx2‐Cre‐ERT2* to induce knockout of conditional MZF1 alleles. Paired *MZF1^fl/fl^*;*Cdx2‐cre‐ERT2* and *MZF1^fl/fl^* littermate control mice were treated with tamoxifen at 8 weeks of age followed by i.p. injection of AOM (10 mg/kg, dissolved in ddH_2_O; Sigma, #A5486) on Day 0. Beginning on Day 5, the mice were administered three cycles of DSS (dissolved in ddH_2_O; Yeasen, #60316ES60) in the drinking water at a concentration of 2.5%. During every cycle, the mice were provided DSS‐containing water for 7 consecutive days followed by regular drinking water for 14 days. Mice were sacrificed on Day 84, and colons were collected and cut open longitudinally to determine the tumor numbers and sizes.

### 4.6 Intestinal Organoid Culture

Fresh primary colon cancer tissue were obtained from *MZF1^fl/fl^*;*Cdx2‐cre‐ERT2* and *MZF1^fl/fl^* mice. Tissues were washed four times with ice‐cold PBS containing penicillin and streptomycin. Each sample was cut into pieces and then digested in the digestion medium for 30 min at 37°C. Digested cells were passed through 100 µm filters and embedded in Matrigel (Thermo Fisher) in wells of 24‐well cell culture plate. Media was added and exchanged every 48 h. Colonic tumor organoids were cultured in Advanced DMEM/F12 (Thermo Fisher) containing 10% HEPES (Thermo Fisher), 1X GlutaMAX (Thermo Fisher), 1X B27 (Thermo Fisher), murine EGF (50 ng/mL, Sigma), murine Noggin (100 ng/mL, PeproTech), 0.5 mm A83‐01 (Tocris), 3 µm SB202190 (Sigma), 1 µm nicotinamide (Sigma), 50% Wnt3A‐conditioned medium, and 20% R‐spondin‐conditioned medium.

### 4.7 CUT&Tag

The CUT&Tag assay was performed using the Active Motif CUT&Tag‐IT Assay Kit. Briefly, fresh cells were collected in Eppendorf (EP) tubes at room temperature and counted. Centrifugation was performed several times to remove supernatant. Cell Perforation Buffer and ConA beads in Binding Buffer were added to the tubes, and the cells were gently inverted and mixed for 5–10 min. Antibody buffer and 0.5–1 µL of primary antibody were added and gently mixed; the EP tube was then incubated on a shaker at room temperature for 2 h. The secondary antibody was diluted 1:100 in Perforation Buffer, added to the tube, and gently shaken. The EP tube was subsequently incubated on a shaker at room temperature for 30–60 min. After incubation, 0.8–1 mL of Perforation Buffer was added, and the tube was inverted up and down 10 times. ChiTag Transposase was diluted 1:250 in Perforation Buffer 2 and added dropwise to the cells; the mixture was incubated for 1 h at room temperature on a shaker, followed by the addition of 1 mL of Perforation Buffer 2, with the tube inverted up and down 10 times. The Tn5 transposase was activated by the addition of a magnesium‐containing reaction solution, which cleaved the DNA region bound by the target protein while simultaneously ligating the carry‐over library junctions to the DNA fragments. DNA was extracted, and PCR amplification was employed to construct the library. High‐throughput sequencing was contracted to Shanghai Jiayin Biotechnology Co.

### 4.8 Chromatin Immunoprecipitation (ChIP)‐qPCR

ChIP was performed using the ChIP‐IT Express Enzymatic Kits (Active Motif) according to the manufacturer's instructions. Cells were fixed with 1% formaldehyde for 10 min, and the reaction was terminated with glycine. The fixed cells were then placed in pre‐cooled PBS. Chromatin fragments were prepared through enzymatic digestion, and the reaction was halted with EDTA. A small aliquot of chromatin was collected to assess breakage and measure concentration. The sheared chromatin was pre‐conjugated to Protein G magnetic beads, and after 2 h, each mixture of chromatin and Protein G magnetic beads was incubated with 4 µg of anti‐Flag antibody (CST, #14793S, RRID: AB_2572291) or normal rabbit IgG (Active Motif, #39087, RRID: AB_2798770) overnight at 4°C. The Protein G beads with antibody‐bound DNA complexes were collected by centrifugation. DNA was eluted, crosslinking reversed, and treated with proteinase K. DNA enriched by ChIP was assessed by quantitative PCR (qPCR). Primer sequences for ChIP analysis are provided in Table S3.

### 4.9 Surface Plasmon Resonance (SPR)

The activator was prepared by mixing 400 mm EDC and 100 mm NHS immediately prior to injection. The CM5 sensor chip was activated for 420 s with the mixture at a flow rate of 10 µL/min. MZF1 was diluted to 20 µg/mL in immobilization buffer and injected into the sample channel (Fc2) at a flow rate of 10 µL/min, which typically resulted in immobilization levels of 3900 RU, the reference channel (Fcl) did not require a ligand immobilization step. The chip is deactivated by 1 M Ethanolamine hydrochloride at a flow rate of 10 µL/min for 420 s. 10Panx Acetate was diluted with the same analyte buffer to six concentrations (200, 100, 50, 25, 12.5 and 0 µm) and injected to channel Fc1‐Fc2 at a flow rate of 30 µL/min for an association phase of 120 s followed by 300 s dissociation. The association and dissociation process were carried out in the analyte buffer. Six cycles were carried out according to analyte concentrations in ascending order. After each cycle of interaction analysis, the analyte will dissociate naturally.

### 4.10 Virtual Screening

The amino acid sequence of MZF1 was downloaded from the National Center for Biotechnology Information (NCBI) and its 3D structure was predicted using the artificial intelligence model AlphaFold 3 (AF3). We found that there are currently no specific reports on the active sites of the MZF1 protein. Therefore, through predictive analysis, we selected two cavities and one potential active‐related site in MZF1 that may be associated with protein activity, along with a known crystallographic fragment, for virtual screening (Figure 6b). Using Schrödinger molecular docking simulation software, the docking grid box center and docking grid box were set up, and a ligand database (in SMILES format) was obtained from Shanghai Taosu Biotechnology Co., Ltd. Based on the ascending order of docking scores, compounds with the strongest binding affinity and multiple interacting residues were selected, and the interactions between the protein and ligands were further analyzed.

### 4.11 Statistical Analysis

Statistical analysis was conducted using GraphPad Prism 9 software (RRID:SCR_002798). The log‐rank test was used for survival analysis. Normally distributed data were expressed as the mean ± standard deviation and analyzed using Student's *t*‐test. Correlation analysis was performed using Pearson's correlation coefficient. One‐way ANOVA was utilized for comparisons among more than two patient groups. A *p*‐value of less than 0.05 was considered indicative of a statistically significant difference.

## Author Contributions

Z.L., X.C. and M.H. conceived the study, designed, performed and interpreted the experiments. Most of the experiments were performed by S.S., Y.W. and M.H. H.W., K.X. and J.C. developed and performed bioinformatics and statistical analysis. S.S. wrote the paper. H.F. and X.B. generated the mouse model. Z.L. and M.H. revised the paper.

## Conflicts of Interest

The authors declare no conflicts of interest.

## Supporting information




**Supporting File 1**: advs77277‐sup‐0001‐SuppMat.docx.

**Supporting File 2**: advs77277‐sup‐0002‐S1‐14.xlsx.

**Supporting file 3**: advs77277‐sup‐0003‐FiguresS1‐S10.zip.

## Data Availability

The data that support the findings of this study are available from the corresponding author upon reasonable request.
