# Supplementary material for: MZF1 Inhibits TGFβ‐1/SMAD Signaling by Transcriptionally Activating SnoN to Augment Tumorigenic Potential in Colon Cancer
Source: Adv Sci (Weinh). 2026 Oct 20:e77277. Online ahead of print. doi: 10.1002/advs.77277 (PMC13683878; doi:10.1002/advs.77277)
Supplement: Supplementary file 1 [file ADVS-9999-e77277-s003.docx]

**MZF1 inhibits TGFβ-1/SMAD signaling by transcriptionally activating SnoN to augment tumorigenic potential in colon cancer.**

**Siqi Sheng^1#^, Mengxi Huang^1#, *^, Yiting Wang^1#^, Hongyu Wang^2^, Haimei Feng^2^, Xiaoming Bai^2^, Jie Chen^1^, Ke Xu ^1^, Xiaoyuan Chu^1*^, Zengjie Lei^1,2,3,4,5*^**

**Supplementary material & methods**

**Plasmids construction, transfection, and lentivirus infection**

MZF1 and SnoN overexpression plasmids were constructed using the full-length CDS from NCBI (human, NM_003422.2 and human, NM_005414), with sequence encoding 3X FLAG and HA tags added to the 3’ end to generate C-terminal tagged fusion proteins, respectively. Lentiviral plasmid construction and viral packaging were carried out by Guangzhou Patson Biotechnology Co and Shanghai Genechem Co. Cells were cultured with the supernatant containing lentivirus and subsequently selected with puromycin, neomycin, or geneticin to obtain the stable expression cell lines. Short hairpin RNA (shRNA) was generated to specifically target the MZF1 and SnoN genes, and lentiviral plasmid construction and viral packaging were carried out by Guangzhou Patson Biotechnology Co and Shanghai Genechem Co.. The interfering sequences are listed in Supplementary Table 1.

**Immunoblotting**

RIPA lysis buffer (KeyGEN Bio TECH, China, #KGB5204-100), supplemented with protease and phosphatase inhibitors (NCM Biotech, China, #P002), was applied to CRC cells or tissues, and the insoluble precipitate was removed by centrifugation. Protein samples were subsequently separated by sodium dodecyl sulfate-polyacrylamide gel electrophoresis (SDS-PAGE) and transferred to a 0.2 µm polyvinylidene difluoride (PVDF) membrane (Millipore, Ireland, #ISEQ00010). Following blocking with 5% non-fat milk, the PVDF membrane was incubated overnight at 4°C with primary antibodies: anti-MZF1 (Abclonal, #A10356, 1:1000, RRID: AB_2757901), anti-SnoN (CST, #4973S, 1:1000, RRID: AB_2082987), anti- PHLDA3 (CST, #4294S, 1:1000, RRID: AB_2799151), anti-CUL4B (GeneTex, #GTX129298, 1:1000, RRID: AB_2885961), anti-SOX10 (CST, #89356 1:1000, RRID: AB_2801056), anti-NFIC (CST, #11911 1:1000, RRID: AB_2798441), anti-SPI1 (CST, #2266 1:1000, RRID: AB_2077789), anti-SMAD2/3 (CST, #8685T 1:1000, RRID: AB_2798840), anti-P-SMAD2/3 (CST, #8828S 1:1000, RRID: AB_2799092), anti-GAPDH (Proteintech, #10494-1-AP, 1:2000, RRID: AB_2107436), anti-β-tubulin (Proteintech, #66240-1-Ig, 1:5000, RRID: AB_11215182) , and anti-β-Actin (Proteintech, #66009-1-Ig, 1:5000, RRID: AB_2242334). The blots were then incubated with secondary antibodies for 1 hour at 37°C. After thorough washing, the blots were visualized using blot detection reagents.

**Quantitative reverse transcription PCR (RT-qPCR)**

Total RNA was isolated from colon cancer cell lines using TRIzol reagent (Invitrogen) and subsequently treated with DNase to eliminate any contaminating DNA. The RNA was reverse transcribed into complementary DNA (cDNA) using reverse transcription reagents (Accurate Biology, #AG11706), and the relative mRNA levels were quantified using a SYBR Green Pro Taq HS Premixed qPCR kit (Accurate Biology, #AG11702). The fold changes of the target genes were normalized to β-actin. Primer sequences for quantitative PCR (qPCR) are provided in Supplementary Table 2. All experiments were performed in triplicate to ensure statistical validity.

**Luciferase reporter gene assay**

HCT8 cells were planted on 12-well plates. When the cell density was approximately 60%, indicated plasmids were transfected for 24 h. β‐Gal was determined by using a β‐gal assay kit (Beyotime) and served as an internal control. Specific experimental details followed the protocol of the Dual-Luciferase Reporter Assay System Kit (Promega).

**Colony formation assays**

Cells were seeded into 6-well plates at 300 or 600 cells/well and cultured for two weeks until the colonies are visible. Colonies were washed with PBS and fixed in 10% methanol for 20 min, then the colonies were stained with 0.4% crystal violet in 20% ethanol for 20 min. Plates were washed in H_2_O and air-dried, and colonies were counted. Three independent experiments were performed to generate mean and standard deviation (S.D.).

**Cell viability assays and 50% inhibitory concentration (IC50) detection**

Cell viability was determined using a CCK-8 assay kit (Biosharp, BS350A). Briefly, CRC cells (transfected or controls) were seeded into 96-well plates at 2,000 cells/well in 200 µL and cultured at 37°C. After cells adhered to the bottom of the well, 10 µl of CCK-8 solution was added to each well. Absorbance at 450 nm wavelength was measured in a microplate reader (Detie, HBS-1096A, China) at 24, 48, 72, and 96 hours.

For IC50 determination, HCT8 and SW480 cells in logarithmic growth phase were seeded in 96-well plates at a density of 6,000 cells/well. After cells adhered to the plate, indicated drugs were added according to a concentration gradient in triplicate. Control wells were treated with the same amount of solvent-containing medium. After 48 hours of incubation, a 100 μL mixture serum-free medium and CCK8 in a 9:1 ratio was added to each well. After 1.5 hours of incubation in a cell culture incubator, the absorbance of each well was measured at a wavelength of 450 nm using an automated microplate reader (Nanbei Instruments), WD-2102A). Cell viability and IC50 values are calculated based on absorbance. Cell viability (%)=(experimental group-blank group)/(control group-blank group)x100

**Transwell assay**

For the transwell assay, 1x10^5^ cells in 200 µL of serum-free medium were placed in the upper chamber (Corning, New York, USA, #3422), while the lower chamber was supplemented with complete medium to assess cell migration. After 24 hours, the chambers were washed twice with PBS, and the cells on the upper membrane were carefully removed. The chambers were then fixed in 4% paraformaldehyde for 20 minutes, washed twice with PBS, and subsequently immersed in 1% gentian violet solution for 30 minutes for staining. The invaded cells were observed in three randomly selected fields using an inverted microscope.

**Immunofluorescence staining**

An appropriate number of cells was seeded into chamber slides. After 24 hours, cells were washed three times with phosphate-buffered saline (PBS) and fixed in 4% paraformaldehyde for 15 minutes. The cells were then washed three times with PBS, permeabilized with 0.5% Triton X-100 for 20 minutes at room temperature, and subsequently washed three times with PBS. Blocking was performed using goat serum for 30 minutes at room temperature, followed by incubation with primary antibodies MZF1 (Abcam, #64866, 1:100, RRID: AB_2457857) SnoN (Thermo Fisher, #MA5-26386 1:150, RRID: AB_2723661) at 4°C overnight. After three washes with PBS, slides were incubated in fluorescent-tagged secondary antibodies (Cy3 Proteintech, #SA00009-1 and Alexa Fluor 488, Jackson ImmunoResearch Laboratories, Inc, #111-545-144) for 1 hour at room temperature in the dark. Subsequently, the cells were incubated with DAPI (Beyotime, #C1006) for 5 minutes at room temperature in the dark to stain the nuclei. Excess DAPI was removed, and the coverslips were sealed with an anti-fluorescence quencher (Beyotime, #P0126). Images were acquired with a fluorescence microscope.

**Bioinformatics**

For the analysis of differentially expressed genes regulating CRC development, we utilized the GEO database (<https://www.ncbi.nlm.nih.gov/geo/query/acc.cgi>) to obtain the raw CEL files of CRC mRNA expression profiling microarrays from three data platforms: GPL570, GPL5175, and GPL96. This included ten datasets: GSE33113, GSE18105, GSE20916, GSE21510, GSE32323, GSE4107, GSE24551, GSE29638, GSE41258, and GSE49355, encompassing a total of 1,215 CRC samples or paraneoplastic tissue samples, which included 1,013 tumor cases and 202 paraneoplastic cases.

The affy package (RRID:SCR_012835) of R software ([http://www.bioconductor.org](http://www.bioconductor.org/)) was utilized to read the raw CEL files. The annotate package was employed to annotate the gene chips, allowing for the identification of genes common to the three platforms. Additionally, the sva package was used to mitigate the batch effect across different platforms, resulting in a total of 1,215 cases. Ultimately, the expression levels of 10,135 genes across these cases were obtained.

For the differential analysis of mRNA gene expression, the limma package (RRID:SCR_010943) was used. The differential gene screening criteria were set as a fold change (tumor tissue/normal tissue) greater than 1.5 (or less than 0.66) with a *P*-value of less than 0.05. The results of the differential analysis were visualized using a volcano plot created with the ggplot2 package (RRID:SCR_014601), while the heatmap package was utilized to visualize the top 100 most significantly upregulated or downregulated genes in tumor tissues.

**Survival analysis and MZF1 stratification**

Patients were classified into MZF1-high and MZF1-low groups based on the median immunohistochemical (IHC) scoring value of MZF1 in the entire cohort. Kaplan–Meier curves were generated for OS, DFS and MFS, and compared by the log-rank test. Univariate and multivariate Cox proportional hazards models were used to assess the independent prognostic value of MZF1 expression, with adjustment for age, gender, TNM stage, tumor location, and histological grade. The proportional hazards assumption was verified graphically and by Schoenfeld residuals.

**Supplementary Figure legend**

**Supplementary Figure S1. MZF1 as a critical transcription factor involved in the malignant progression of colorectal cancer.**

A. Venn diagram of the number of genes included in the GEO database for colorectal cancer across three platforms.

B-C. Kaplan-Meier survival analysis of MZF1 expression level in tumor tissue and prognosis of patient disease-free survival (DFS) (b) and metastasis-free survival (MFS) (c). Blue represents the low expression group of MZF1 (n=60), and red represents the high expression group of MZF1 (n=36).

D. Immunoblotting analysis of protein levels of SPI1, SOX10, and NFIC in tumor tissue (T) and normal adjacent tissue (A) of clinical specimens. GAPDH is used as an internal reference.

**Supplementary Figure S2. MZF1 promotes colon cancer cell proliferation *in vitro*.**

A. Immunoblot of MZF1 protein in CRC cell lines.

B-C. Protein (b) and mRNA (c) levels of MZF1 in SW480 cells infected with control or MZF1 overexpression lentivirus and mock infected cells using immunoblotting (b) and RT-qPCR (c).

d-e. Protein (b) and mRNA (c) levels of MZF1 in HCT15 cells infected with shControl or shMZF1 expressing lentivirus and mock infected cells using immunoblotting (d) and RT-qPCR (e).

F-G. Representative images of colony formation assays (f) and soft agar assays (g) of SW480 cells infected with control or MZF1 overexpression lentivirus and mock infected cells.

H-I. Transwell assays of HCT8 cells (h) and SW480 cells (i) infected with control or MZF1 overexpression lentivirus and mock infected cells.

j-k. Colony formation assays (j) and soft agar assay (k) of HCT15 cells infected with shControl or shMZF1 lentivirus and mock infected cells.

All assays were performed in triplicate and *P* values were calculated using two-tailed, unpaired Student’s *t*-test.

**Supplementary Figure S3. MZF1 is required for CRC tumor growth *in vivo*.**

A-B. PCR-based genotyping analysis for *MZF1^fl/fl^* (a) *Cdx2-cre-ERT2* (b) alleles.

C. Representative images of hematochezia of mice in both *MZF1^fl/fl^* and *MZF1^fl/fl^*;*Cdx2-cre-ERT2* mice.

D. Body weight curves of *MZF1^fl/fl^* and *MZF1^fl/fl^*;*Cdx2-cre-ERT2* mice during the AOM/DSS-induced orthotopic CRC model.

e. Colon length of *MZF1^fl/fl^* and *MZF1^fl/fl^*;*Cdx2-cre-ERT2* mice following completion of the AOM/DSS-induced orthotopic CRC model.

**Supplementary Figure S4. MZF1 transcriptionally activates SnoN in colon cancer.**

A. Heatmap of mRNA levels of differentially expressed genes following RNA-seq analysis of HCT8-NC and HCT8-LVMZF1 cells.

B. Venn diagram of the overlapping genes identified by RNA-seq and aligned to CUT&Tag enriched peaks.

C. GO annotation analyzes of the 2,418 overlapping target genes in panel b that may be directly transcriptionally regulated by MZF1.

D. Correlation analysis of MZF1 and SnoN expression in HCT8 cells. Pearson correlation analysis of single-cell fluorescence intensities demonstrates a significant positive correlation between MZF1 and SnoN levels (Pearson r = 0.7959, P < 0.0001)

**Supplementary Figure S5. MZF1 transcriptionally activates SnoN in colon cancer.**

A-B. Immunoblotting of MZF1, CUL4B, and tubulin in HCT8 (a) and SW480 (b) cells infected with control or MZF1 overexpression lentivirus and mock infected cells.

C-D. RT-qPCR of *MZF1*, *PHLDA3,* and *CUL4B* in HCT8 (c) and SW480 (d) cells infected with control or MZF1 overexpression lentivirus and mock infected cells.

E-F. Immunoblotting of MZF1, CUL4B, and tubulin in HCT8 (e) and SW480 (f) cells infected with shControl or shMZF1 expressing lentivirus and mock infected cells.

G. Immunoblotting of MZF1, PHLDA3, and tubulin in HCT8 cells infected with shControl or shMZF1 expressing lentivirus and mock infected cells.

H-I. RT-qPCR of MZF1, *PHLDA3,* and *CUL4B* in SW480 (h) and HCT8 (i) cells infected with indicated shControl or shMZF1 expressing lentivirus and mock infected cells.

j-k. ChIP-qPCR experiments showing enrichment of MZF1 on the *PHLDA3* (j) and *CUL4B* (k) promoters in HCT8 cells.

l-m. ChIP-qPCR experiments showing enrichment of MZF1 on the *PHLDA3* (j) and *CUL4B* (k) promoters in SW480 cells.

ANOVA was used for multiple comparisons; *P*<0.05 is considered signficant.

**Supplementary Figure S6. MZF1 transcriptionally activates SnoN and regulates TGF-β signaling to promote colon cancer cell proliferation.**

A. Immunoblotting of SnoN and tubulin in CRC cell lines.

B-C. mRNA and protein levels of SnoN were measured by RT-qPCR and immunoblotting, respectively, in RKO (b) or Lovo (c) cells infected with control or SnoN overexpressing lentivirus.

d-e. mRNA and protein levels of SnoN were measured by RT-qPCR and immunoblotting, respectively, in SW480 (d) or HCT8 (e) cells infected with shControl or shSnoN expressing lentivirus or mock infected cells.

F-G. Colony formation assays of RKO (f) and Lovo (g) cells infected with control or SnoN overexpressing lentivirus.

H. Soft agar assays of RKO cells infected with shControl or shSnoN expressing lentivirus or mock infected cells.

i-j. CCK8 assays of RKO (i) and Lovo (j) cells either mock infected or infected with control or SnoN overexpressing lentivirus.

k-l. Colony formation assays of SW480 (k) and HCT8 (l) cells infected with shControl or shSnoN expressing lentivirus or mock infected cells.

m-n. CCK8 assays of SW480 (m) and HCT8 (n) cells infected with shControl or shSnoN expressing lentivirus or mock infected cells.

ANOVA was used for multiple comparisons; *P*<0.05 is considered signficant.

**Supplementary Figure S7. MZF1 transcriptionally activates SnoN and regulates TGF-β signaling to promote colon cancer cell proliferation.**

A-B. Immunoblotting to assess protein levels of MZF1, SnoN, and tubulin in HCT8-shMZF1 (a) or SW480- shMZF1 (b) cells infected with shControl or shSnoN expressing lentivirus or mock infected cells.

C-D. RT-qPCR to assess mRNA levels of MZF1 and SnoN in HCT8-shMZF1 (c) or SW480-shMZF1 (d) cells infected with control or SnoN overexpression lentivirus.

E-F. Representative images of colony formation assays of HCT8-LV-MZF1 (e) or SW480-LV-MZF1 (f) cell lines infected with shControl or shSnoN expressing lentivirus or mock infected cells.

G-H. CCK8 assays of HCT8-shMZF1 (g) or SW480-shMZF1 (h) cell lines infected with control or SnoN overexpression lentivirus.

I. Representative images (left) and quantification (right) of colony formation assays of HCT8-shMZF1-2 cells infected with shControl or shSnoN expressing lentivirus or mock infected cells.

All assays were performed in triplicate. ANOVA was used for multiple comparisons; *P*<0.05 is considered signficant.

J-K. CAGA-luciferase reporter assays evaluating SMAD transcriptional activity in HCT8 (j) and SW480 (k) cells. Cells stably expressing MZF1 (LV-MZF1) or co-expressing MZF1 and shSnoN-2 (LV-MZF1+shSnoN-2) were transfected with the CAGA-luc reporter and Renilla control plasmids, followed by stimulation with TGF-β1 (10 ng/mL).

**Supplementary Figure S8. MZF1 inhibitor 10Panx Acetate suppresses colon cancer cell proliferation *in vitro* and colon cancer tumorigenesis *in vivo*.**

A. Heatmap of SW480 cell survival rate for 15 compounds using cell viability assay.

B. Predicted interaction diagram of MZF1 with dBET6.

C. Chemical structure of dBET6, a bromodomain-containing protein inhibitor.

Schematic diagram of MZF1 three-dimensional structure (left) and MZF1 internal cavity (right).

D. Immunoblotting for MZF1, SnoN, and tubulin in SW40 cells with or without MZF1 overexpression treated in the presence or absence of 10Panx Acetate.

e. CCK8 assays in SW480 cells with or without MZF1 overexpression treated in the presence or absence of 10Panx Acetate.

F. Colony formation assays in HCT8 cells with or without MZF1 overexpression treated in the presence or absence of 10Panx Acetate.

G-H. Representative images (g) and quantification (h) of colony formation assays in SW480 cells with or without MZF1 overexpression treated in the presence or absence of 10Panx Acetate.

**I.** CCK8 assays in HCT8 cells with or without MZF1 knockdown (shMZF1-2) treated in the presence or absence of 10Panx Acetate.

**J-K.** Representative images (J) and quantification (K) of colony formation assays in HCT8 cells with or without MZF1 knockdown treated in the presence or absence of 10Panx Acetate.

L-M. CCK8 assays in HCT8 (i) or SW480 (j) cells with or without MZF1 overexpression treated in the presence or absence of dBET6.

N-O. Representative images (k) and quantification (l) of colony formation assays in HCT8 cells with or without MZF1 overexpression treated in the presence or absence 27of dBET6.

P-Q. Representative images (m) and quantification (n) of colony formation assays in SW480 cells with or without MZF1 overexpression treated in the presence or absence of dBET6.

All assays were performed in triplicate. ANOVA was used for multiple comparisons; *P*<0.05 is considered signficant.

**Supplementary Figure S9. MZF1 inhibitor 10Panx Acetate suppresses colon cancer cell proliferation *in vitro* and colon cancer tumorigenesis *in vivo*.**

A. Relative mRNA expression levels of *MZF1* and *SnoN* in HCT8-LV-MZF1 cells treatment with DMSO or 10Panx Acetate.

B. Representative immunoblots (left) and quantification (right) of SW480 cells treated for the indicated times with cycloheximide (CHX) (100 μg/ml) in the presence or absence of 10Panx Acetate.

c-e. Relative cell viability measured by CCK8 assay in HCT8 cells treated with partial sequences of dBET6: Sequence 1 (D{D-SER}{D-TYR}) (C), Sequence 2 (WRQ) (D), or both combined (E).

F. Relative mRNA expression levels determined by RT-qPCR of downstream targets of dBET6 inhibitor (*c-myc*, *BRD4*, *HGF*, *ISG15*, and *LAMB*) in HCT8 cells with or without MZF1 overexpression treated in the presence or absence of dBET6.

G. Body weight curves of mice bearing xenografts either mock or overexpressing MZF1 (LV-MZF1) cells treated with 10Panx Acetate (10mg/kg) or saline. (n=6 per group)

H. H&E staining of heart, liver, spleen, lung, kidney and brain tissues from mice treated in panel G.

Unless indicated otherwise all assays were performed in triplicate. ANOVA was used for multiple comparisons; *P*<0.05 is considered signficant.

**Supplementary Figure S10. MZF1 and SnoN are both overexpressed in colon cancer tissues, and their co-overexpression correlates with poor prognosis.**

A. TCGA datasets were analyzed to examine the expression of *SnoN* in READ samples (n=166) and normal tissues (n=10).

B. GTEx datasets were analyzed to examine the expression of *SnoN* in READ samples (n=92) and normal tissues (n=318).

C. Immunoblotting of MZF1, SnoN, and tubulin in tumor tissue (T) and adjacent normal tissue (A) of clinical tissue specimens.

D. Correlation analysis of MZF1 and SnoN expression in cohort CRC tissues (n=76).

e. Correlation analysis of MZF1 IHC scores and SnoN IHC scores.

F. Kaplan-Meier survival analysis of SnoN^low^ (n=41) and SnoN^high^ (n=35) expression level in tumor tissue and patient MFS prognosis.

G-H. IHC scoring of MZF1 (f) and SnoN (g) in adjacent normal tissue (n=83) and tumor tissues (n = 83) in the SOB cohort.

I-K. Representative IHC images (h) and IHC scoring of MZF1 (i) and SnoN (j) in adjacent normal tissue (n=80) and tumor tissues (n = 80) in the AFB cohort. Scale bar: 20 μm.

L. Spearman correlation analysis of MZF1 and SnoN IHC scores in tumor tissues in AFB cohort (n= 80).

M-O. Kaplan-Meier survival analysis of low versus high expression of MZF1 (l), SnoN (m) or MZF1 and SnoN (n) expression level and patient OS prognosis in AFB cohort.

**Supplementary Table catalogue**

Supplementary Table S1 Interfering sequences.

Supplementary Table S2 Primers for qPCR analysis.

Supplementary Table S3 Primers for ChIP analysis.

Supplementary Table S4 GEO database colorectal cancer mRNA expression profiling microarray.

Supplementary Table S5 All differentially expressed genes between tumor tissues and normal tissues in colorectal cancer mRNA expression profiling microarrays.

Supplementary Table S6 Transcriptional factor co-regulation analysis

Supplementary Table S7 Correlation analysis of MZF1 expression levels with clinicopathological data

Supplementary Table S8 Univariate and multivariate Cox regression analysis of clinicopathological factors with OS

Supplementary Table S9 RNA-seq library.

Supplementary Table S10 HCT8-LV-MZF1 CUT&Tag library.

Supplementary Table S11 The 9,442 genes obtained through the genemerge of the intersection from two CUT&Tag sequencing results of HCT8-LV-MZF1 cells.

Supplementary Table S12 The 2,418 genes obtained through the genemerge of the intersection between RNA sequencing and CUT&Tag sequencing results.

Supplementary Table S13 15 potential inhibitors identified by virtual screnning

Supplementary Table S14 Univariate and multivariate Cox regression analysis of risk factors for OS in two cohorts.
